# A Comprehensive Review on Advanced Extraction Techniques for Retrieving Bioactive Components from Natural Sources

**DOI:** 10.1021/acsomega.4c02718

**Published:** 2024-07-08

**Authors:** Yogesh A. Bhadange, Jitendra Carpenter, Virendra Kumar Saharan

**Affiliations:** † Department of Chemical Engineering, Malaviya National Institute of Technology, Jaipur 302017, India; ‡ Department of Chemical Engineering, Manipal Institute of Technology, Manipal Academy of Higher Education (MAHE), Manipal 576104, India

## Abstract

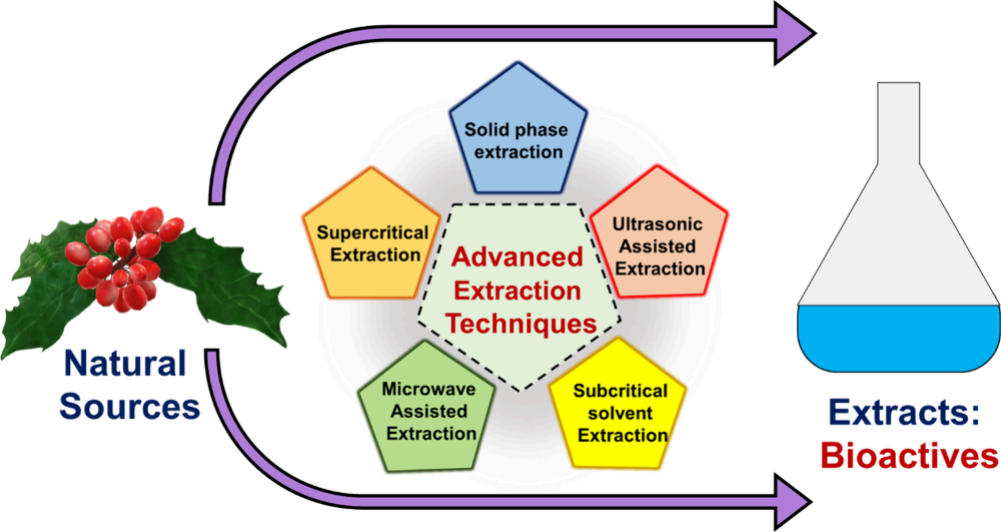

The extraction of bioactive components from natural sources has gained significant attention in recent years due to increasing demand for natural and functional constituents in various industries, including pharmaceuticals, food, and cosmetics. This review paper aims to provide a comprehensive overview of the studies on extracting bioactive components from natural sources using different advanced extraction techniques. It highlights the need for efficient extraction methods to preserve these components’ integrity and bioactivity. Various extraction techniques as supercritical-fluid extraction, microwave-assisted extraction, ultrasound-assisted extraction, subcritical solvent extraction, and solid-phase microextraction are explored in detail, highlighting their principles, advantages, and limitations. The review further examines the impact of different factors on the extraction process, including solvent selection, extraction time, temperature, ultrasonication-amplitude, etc. Additionally, emerging techniques, such as green extraction methods and nanotechnology-based approaches, are discussed, emphasizing their potential to enhance the extraction efficiency and sustainability of the process. Furthermore, the review presents case studies and experimental results from recent research articles, providing insights into applying different extraction techniques for specific bioactive components, such as phenolics, flavonoids, alkaloids, and essential oils. It discusses the extraction yield, bioactivity, and potential utilization of the extracted components in various industries. Overall, this review paper is valuable for researchers, scientists, and industry professionals interested in extracting bioactive components from natural sources. It consolidates the current knowledge on different advanced extraction techniques, their optimization parameters, and their potential applications, facilitating further advancements in the field and the development of innovative extraction methods for bioactive component extraction from natural sources.

## 1 Introduction

Bioactive components are natural compounds found in various plants, animals, and microorganisms that possess biological activity and have the potential to impact human health positively. These components, such as phytochemicals, antioxidants, vitamins, and more, are vital for wellness and disease prevention, relevant to medicine, nutrition, and pharmacology.^1^ Extensive research has shown that bioactive components exhibit various biological activities, such as antioxidant, anti-inflammatory, antimicrobial, anticancer, and neuroprotective properties.^2^ These activities can help combat oxidative stress, reduce chronic inflammation, enhance the immune system, inhibit the growth of cancer cells, and protect against age-related cognitive decline. Moreover, bioactive components have been linked to the prevention and management of several chronic diseases, including cardiovascular diseases, diabetes, obesity, and neurodegenerative disorders.^2^

These components are often used to formulate products that target specific health conditions or promote general well-being. Some of the bio active components are beta-carotene (source: carrots, sweet potatoes, spinach; benefits: converts to vitamin A, supports vision, antioxidant.),^1^ berberine (source: goldenseal, barberry, oregon grape; benefits: antimicrobial, anti-inflammatory, blood sugar regulation),^2^ allicin (source: garlic; benefits: antimicrobial, cardiovascular support, immune system boost),^3^ salicylic acid (source: willow bark, meadowsweet; benefits: anti-inflammatory, pain relief, skincare.),^4^ fisetin (source: strawberries, apples, onions; benefits: antioxidant, anti-inflammatory, potential antiaging),^5^ echinacea purpurea (Source: echinacea plant; benefits: immune system support, anti-inflammatory),^6^ betaine (source: beets, spinach, whole grains; benefits: liver health, cardiovascular support, cognitive function),^7^ etc. Bioactive components hold immense importance due to their potential to improve human health and contribute to the development of innovative and natural-based therapeutic approaches. Further research and understanding of these components are essential for harnessing their full potential and promoting a healthier lifestyle.^2^

The extraction of bioactive components from natural sources is crucial in obtaining their desired properties and maximizing their potential applications. Various advanced extraction techniques have been developed over conventional methods to efficiently isolate and concentrate these valuable components from their sources.^3^ Conventional extraction techniques involve traditional methods like maceration, distillation, and Soxhlet extraction, whereas advanced extraction techniques includes supercritical fluid extraction, ultrasound-assisted extraction, microwave-assisted extraction, etc. The importance of advanced techniques lies in their ability to enhance efficiency, reduce extraction time, and minimize solvent usage, leading to higher yields of bioactive components.^3^ Moreover, advanced methods often result in improved selectivity and preservation of delicate compounds, ensuring the extraction of high-quality and potent bioactives. These techniques also facilitate environmental friendly processes by reducing the use of harmful solvents. Overall, the significance of advanced extraction techniques lies in their capacity to optimize extraction parameters, increase productivity, and yield of bioactive components with enhanced purity and bioavailability, making them crucial for applications in pharmaceuticals, nutraceuticals, and other industries.^3^

Supercritical fluid extraction (SFE) is a technique that utilizes supercritical fluids, such as carbon dioxide, to extract bioactive components. SFE offers advantages like low toxicity, minimal residue, and tunable solvent power. Microwave-assisted extraction (MAE) utilizes microwave energy to accelerate the extraction process, while ultrasound-assisted extraction (UAE) employs high-frequency ultrasound waves to enhance mass transfer and disrupt cell structures, leading to increased extraction efficiency. Green extraction techniques have gained prominence in recent years due to their sustainable and environmental friendly nature. These methods include subcritical solvent extraction and solid-phase microextraction. Subcritical solvent extraction (SSE) employs solvent at temperatures and pressures below its critical point, enhancing the extraction of bioactive compounds from various materials due to its unique solvation properties, while solid-phase microextraction (SPME) is a solvent-free sampling technique that efficiently extracts and concentrates analytes, reduces the use of hazardous solvents, and minimizes waste generation while maintaining high extraction yields. Furthermore, nanotechnology-based approaches have emerged as promising extraction methods that enhance solubility, stability, and bioavailability, leading to improved extraction efficiency.

The choice of extraction technique depends on factors such as the nature of the bioactive components, the matrix complexity, target yields, and the desired application. Optimizing extraction parameters like solvent selection, extraction time, temperature, ultrasonic amplitude, pressure, power, etc. can significantly impact the extraction efficiency and quality of the obtained bioactive components.^4^ Hence, selecting an appropriate extraction technique is crucial for obtaining desired bioactive components from natural sources. The advancement and utilization of these bioactive constituents within the pharmaceuticals, food, and cosmetics sectors contribute to the augmentation of product quality, functionality, and intrinsic value.^5^

The principle objective of this review study was to highlight the potential applications of the bioactive components and to provide an overview of the advanced extraction techniques used for their extraction from natural sources. Discuss recent case studies and experimental results to showcase the effectiveness of different advanced extraction techniques for specific bioactive components. Explore the principles, advantages, and limitations of different extraction techniques. Investigate the influence of various extraction parameters on the efficiency and yield of bioactive component extraction. Identify emerging trends and recent advancements in the field of bioactive component extraction. Facilitate the development of innovative and sustainable extraction methods for bioactive components from natural sources. The current review serves as a valuable resource for researchers, scientists, and industry professionals interested in bioactive component extraction and its applications.

### 1.1 History

The history of advanced extraction methods is rooted in the quest for more efficient and precise techniques to extract valuable compounds from natural sources. Traditional extraction methods, relying on simple processes like maceration, distillation, Soxhlet extraction, decoction, etc. often proved time-consuming and inefficient.^4^ The need for improved methodologies led to the development of advanced techniques. Supercritical fluid extraction (SFE) emerged as a significant advancement in the 20^th^ century, utilizing supercritical fluids like carbon dioxide to enhance extraction efficiency.^5^ This method gained traction in the pharmaceutical and food industries for its ability to selectively extract compounds without leaving behind solvent residues. In the late 20^th^ century, ultrasound-assisted extraction (UAE) gained prominence. Initially used in analytical chemistry, UAE proved effective in breaking cell walls and accelerating mass transfer, leading to faster and more efficient extractions.^5^ This method found applications in various industries, from pharmaceuticals to environmental analysis. Microwave-assisted extraction (MAE) also became prominent during the late 20^th^ century.^6^ Leveraging microwave energy, this technique facilitated rapid and selective extraction of compounds, particularly in the fields of herbal medicine and natural product synthesis. The 21^st^ century witnessed further innovation with the integration of technologies like nanotechnology and green chemistry principles into extraction methodologies.^6^ These advancements aimed to enhance sustainability, reduce environmental impact, and improve the scalability of extraction processes. As demand grew for natural products in pharmaceuticals, nutraceuticals, and cosmetics, researchers continually sought innovative ways to extract and concentrate bioactive compounds.^7^ Advanced extraction methods became pivotal in meeting these demands, offering solutions that were not only efficient but also environmentally friendly. The ongoing evolution of extraction methods reflects a dynamic intersection of scientific, technological, and industrial advancements, with a focus on optimizing processes for a sustainable and resource-efficient future.

## 2 Advanced Extraction Techniques

Advanced extraction techniques have revolutionized the extraction field, enabling more efficient and selective extraction of desired compounds from diverse sources. These advanced techniques offer higher selectivity, improved yields, reduced solvent usage, and faster extraction times, catering to the growing demands of various industries, including pharmaceuticals, food, and environmental analysis.

### 2.1 Microwave-Assisted Extraction

**Figure 1:**
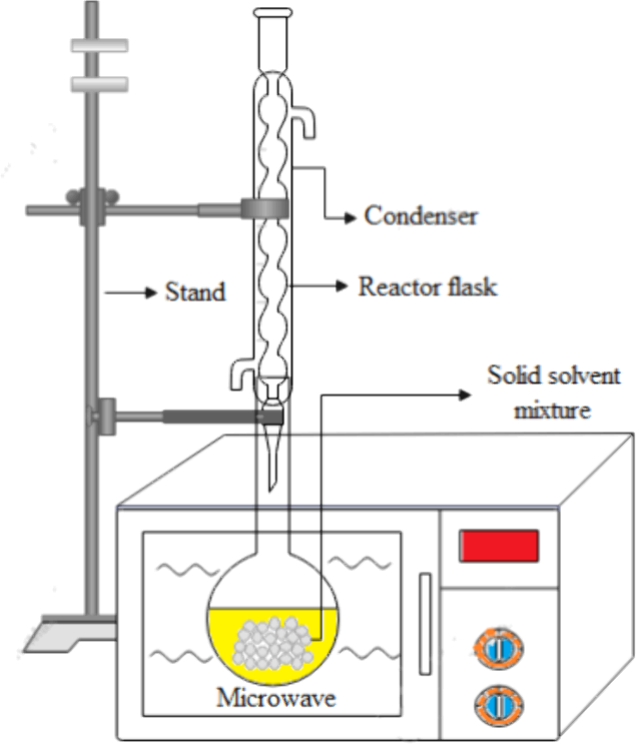
Diagram of microwave assisted extraction.

Microwave-assisted extraction (MAE) is a modern extraction technique that utilizes microwave energy to enhance the extraction process. It is widely used in various industries, including pharmaceuticals, food, and natural product extraction.^12^ In MAE, the sample material is mixed with a suitable solvent in an extraction vessel. Microwave energy is then applied, which rapidly heats the mixture, causing the solvent to boil and creating internal pressure within the sample. This pressure helps to rupture the cell walls and facilitate the extraction of target compounds.^12^ A detailed and technical illustration of the microwave-assisted extraction (MAE) process is shown in Figure 1.

The extraction vessel is a microwave-safe container that holds the sample material and solvent mixture. It is typically made of glass or other suitable materials that can withstand microwave radiation. The sample material is usually finely ground or chopped to increase the surface area available for extraction. It is then mixed with a suitable solvent in the extraction vessel. The solvent choice depends on the nature of the target compounds and their solubility. The extraction vessel is placed under microwave irradiation, which generates and delivers microwave energy to the solution. The applicator ensures uniform and controlled heating of the entire extraction vessel. Microwave power levels can vary depending on the specific application and the characteristics of the sample. When the microwave energy is applied, the solvent absorbs the energy and rapidly heats up. The heat causes the solvent to boil and creates internal pressure within the sample, leading to the rupture of cell walls and the release of target compounds.^13^ To enhance mass transfer and ensure uniform heating, the sample may be stirred or mixed during the extraction process. This promotes efficient extraction by facilitating the contact between the solvent and the substrate molecules. Parameters such as microwave power, temperature, and extraction time may require optimization based on the sample characteristics and the desired compounds. Safety precautions must be followed when performing microwave-assisted extraction, as microwave radiation can be hazardous.^13^ Adhering to proper procedures and guidelines is essential to ensure the safe and effective operation of the MAE process.

#### 2.1.1 Key Parameters for Efficiency and Yield Enhancement in MAE

##### 2.1.1.1 Temperature

In MAE, temperature plays a pivotal role in enhancing the effectiveness of extraction of bioactive compounds from plant materials. Elevated temperatures facilitate the rupture of cell walls, promoting the release of target compounds. In the study on MAE of neem oil by Nde et al.,^8^ temperature played a critical role in optimizing the process. The temperature range was adjusted between 50 and 100 °C to assess its impact on the extraction yield. The determined optimum conditions were heating time of 34.43 min, heating temperature of 79.21 °C, and solvent/solute ratio of 2.95 for oil content. These conditions led to a substantial increase in oil yield (31.65% w/w) compared to traditional Soxhlet extraction, which required 10 h. Gunalan et al.,^9^ reported the extraction of gallic acid from moringa oleifera in the presence of MAE at temperature in the range of 30–50 °C. At 40 °C and 600 W power, the study recorded a maximum extraction yield of 17.65% (w/w) with total phenol content of 76.40 mg/g. Beyond this temperature, a decline in both extraction yield and phenolic compounds occurred, attributed to thermal degradation and oxidation. The importance of precise temperature control in MAE was evident, highlighting the delicate balance between maximizing extraction efficiency and avoiding detrimental effects on heat-sensitive compounds.^9^ Controlled temperatures optimize the interaction between the solvent and matrix, ensuring efficient extraction while minimizing thermal degradation. Additionally, temperature influences the viscosity and diffusivity of the solvent, impacting mass transfer kinetics. Proper temperature management in MAE enhances extraction yield, reduces processing time, and preserves the integrity of extracted compounds, making it a crucial parameter for achieving optimal results in this innovative and sustainable extraction technique.^8^

##### 2.1.1.2 Extraction Time

Extraction time is a pivotal factor in MAE has deep implications on the efficiency and selectivity of compound extraction. The extent of exposure to microwave energy directly influences the breakdown of cell structures, facilitating the release of bioactive components from plant matrices. Nde et al.,^8^ studied the effect of time on the extraction of neem oil. The determined optimal parameters included a heating duration of 24 min, a heating temperature of 79.21 °C, and a solvent-to-solute ratio of 2.95 (mL/g) for oil content. The quantity of oil extracted under these conditions was 31.65% (w/w) at an optimum condition. Notably, there was no significant increase in the oil yield beyond 24 min of extraction, highlighting the efficiency of the shorter extraction time. The retained optimum conditions emphasized a lower extraction time (24 min), showcasing a substantial gain in reaction time. This time-efficient approach, coupled with the reduced acid value compared to Soxhlet extraction, underscores the potential of MAE for rapid and cost-effective neem oil extraction, addressing the increasing demand in various industries.

Gunalan et al.,^9^ investigated the effect of time on the extraction of gallic acid from Moringa Oleifera using MAE. The study revealed that at an optimum extraction time of 30 min, the maximum extraction yield obtained was 17.65% (w/w) with a total phenol content of 76.40 mg/g.^9^ On the contrary, in the study reported by Chew et al.,^10^ on the extraction of rutin from female carica papaya linn leaves. The results indicated that extraction time exhibited a generally less significant impact on rutin yield across all methods studied. The optimal condition for MAE was determined to be 9.3 min, resulting in a rutin yield of 5.67 ± 0.16 mg/g. Despite its comparatively lower impact, the extraction time remains a crucial parameter influencing the efficiency of rutin extraction processes. Balancing extraction time with other parameters is essential to achieve optimal rutin yields while considering resource efficiency and energy consumption in the context of sustainable extraction methodologies. Optimal extraction time ensures sufficient interaction between the solvent and the sample, promoting higher yields of target compounds. However, excessively prolonged extraction times may lead to the degradation of sensitive compounds. Therefore, cautious control of extraction time in MAE is crucial for achieving maximum extraction efficiency while preserving the integrity of the extracted bioactive substances.^9^

##### 2.1.1.3 Solid to Solvent Ratio (SS Ratio)

The solid to solvent ratio (SS ratio) in MAE significantly influences the competence of bioactive compound extraction. An optimal ratio enhances the solubility of components, promoting efficient release from the matrix. In the study reported by Chew et al.,^10^ the solid-to-solvent ratio was identified as a crucial parameter in the MAE process for rutin extraction from female carica papaya linn leaves. It was observed that on increasing the ratio from 1.10 to 1.170 (w/w), the yield of rutin was increased from 1.8 to 7 mg/g. The study emphasizes that a higher solid to solvent ratio led to a steeper concentration gradient between the leaf sample and the ethanol mixture, facilitating easier diffusion of rutin from the plant matrix into the solvent. Similarly, in the study on MAE of black bean hull powder by Mali et al.,^11^ the effect of solid to solvent ratio on the efficiency of bioactive compound (anthocyanins, phenolics, flavonoids) extraction was investigated. A higher solvent to solid ratio (50:1 mL/g) resulted in the highest yield of bioactive components, showcasing the importance of this parameter in enhancing the extraction efficiency. The study’s numerical optimization identified the optimal conditions for MAE, emphasizing a solvent to solid ratio of 50 mL/g. This optimized condition yielded higher levels of total anthocyanins (33.82 mg/g), total phenolics (200.37 mg/g), and total flavonoids (86.02 mg/g) compared to conventional solvent extraction method. The solid to solvent ratio had not only influenced the solubility of components but also facilitated efficient anthocyanin and flavonoid release from the matrix.^10^ This ratio significantly influences the interaction between the solvent and the matrix, affecting mass transfer rates and ultimately enhancing the extraction of valuable components. Optimizing the solid-to-solvent ratio is crucial for achieving maximal extraction efficiency, ensuring the economical use of solvents, and producing extracts with high concentrations of bioactive compounds.^11^ Optimization of this parameter is essential for the scalability and sustainability of MAE processes, underscoring its significance in achieving optimal outcomes for various applications.

##### 2.1.1.4 Microwave Power

Microwave power is a crucial parameter in MAE as it directly impacts the productivity of extracting bioactive compounds from plant materials. The selected microwave power determines the heating rate within the sample, affecting the release of target compounds. Gunalan et al.,^9^ explored the microwave-assisted extraction of gallic acid from Moringa Oleifera and studied the effect on extraction yield at different power in the range of 500 to 700 W. A maximum yield of 17.65% (w/w) was obtained at a power dissipation of 600 W. A notable decrease in extraction yield at elevated power (700 W), suggests a delicate balance for optimal biomolecule recovery is required. The study emphasized that the optimization of microwave power is vital for enhancing the efficiency of MAE and obtaining extracts rich in bioactive components. In another study, Mali et al.,^11^ investigated the impact of microwave power on the extraction efficiency of bioactive compounds from black bean hull powder. The maximum yield in terms of total anthocyanin and phenolic content of 34.14 mg/g and 197.23 mg/g with their respective antioxidant activities of 91.37% and 93.51%, respectively, was obtained at a power dissipation of 344 W. Too low power may result in inadequate extraction, whereas excessively high power can lead to the degradation of sensitive compounds. Therefore, optimizing microwave power is essential for maximizing yield and maintaining the integrity of extracted bioactive substances in MAE. An overview of some of the recent MAE studies reported in the literature is given in Table 1.

**Table 1:** Overview of Advanced Extraction Studies Reported in the Literature

botanical species	sources	bioactive component	extraction method	operating condition	ref
Neem	fruit	oil	MAE	solvent: hexane	(8)
				SS ratio- 30:100 v/w	
				time: 3–10 min	
				temperature: 50–100 °C	
				power: 1000 W	
				yield: 80% (w/w)	
Carica papaya Linn.	leaf	rutin	MAE	solvent: ethanol 20%	(10)
				SS ratio: 1:650 (w/w)	
				time: 20 min	
				temperature: 27 °C	
				power: 100 and 800 W	
				yield: 18.43 ± 0.81 mg/g	
Moringa (Moringa oleifera Lam.)	leaf	biomolecule	MAE	solvent: ethanol 70%	(9)
				SS ratio: 1:10 g/mL	
				time: 20–40 min	
				temperature: 30–50 °C	
				power: 500–700 W	
				yield: 76.40 mg/g	
Black Beans	seed	antioxidant and antidiabetic	MAE	solvent: ethanol: water (100:0–0:100)	(11)
				SS ratio: 20:1 –50:1 w/v	
				time: 2–6 min	
				temperature: 40 °C	
				power: 334.4 W	
				yield: 197.23 mg/g	
Propolis	casein micelles	chrysin	MAE	solvent: ethanol	(14)
				SS ratio: 3:30 w/v	
				time: 15 min	
				temperature: 25 °C	
				power: 100 W	
				yield: 15.81% (w/w)	
Jerusalem	artichoke	phenolics	MAE	solvent: hexane	(15)
				SS ratio: 30:100 v/w	
				time: 11.96 min	
				temperature: 79.18 °C	
				power: 13.49 W	
				yield: 4483.33 mg/kg	
Amorphophallus muelleri	flour	glucomannan	MAE	solvent: ethanol	(16)
				SS ratio: 30:100 v/w	
				time: 5–20 min	
				temperature: 79.18 °C	
				power: 300 W	
				yield: 140.2 mg/g	
Orange	peel	pectin	MAE	solvent: DES	(12)
				SS ratio: 8:100 g/mL	
				time: 15 min	
				temperature: 79.18 °C	
				power: 360 W	
				yield: 7.14 mg/g	
Silybum marianum L.	thistle	triglycerides and flavonolignans	SFE	solvent: pure ethanol from 10% to 40%	(18)
				solid: 1 g	
				temperature: 40 - 60 °C	
				pressure: 15 - 25 MPa	
				time: 30 - 90 min	
				yield: 92–94% (v/v)	
Agaricus bisporus mushroom	fruit	ergosterol	SFE	solvent: CO_2_	(19)
				solid: 4g	
				temperature: 80 °C	
				pressure: 300 bar	
				time: 4 mL/min	
				yield: 547.27 mg/g	
Dendrobium chrysotoxum	flowers	flavonoids	SFE	solvent: ethanol (60%)	(20)
				solid: 1:40 (w/v)	
				temperature: 50 °C	
				pressure: 20 MPa	
				time: 90 min	
				yield: 2.04% (w/w)	
Flaxseed	seed	oil	SFE	solvent: CO_2_	(22)
				oil: 500 mL	
				temperature: 30 °C	
				pressure: 55 MPa	
				time: 120 min	
				yield: 33.66% (w/w)	
Syzygium aromaticum	leaves	eugenol	SFE	solvent: ethanol	(23)
				solid: 7.5 g	
				temperature: 60 °C	
				pressure: 300 bar	
				time: 30 min	
				yield: 8.06 g/kg	
Acacia dealbata	link bark	lupane-triterpenoids	SFE	solvent: ethyl acetate: acetone	(21)
				solid: 7.5 g	
				temperature: - 40–80 °C	
				pressure: - 10–30 MPa	
				time: - 30 min	
				yield: −777.5 mg/kg	
Carob	fruit	polyphenolic composition	UAE	solvent: acetone (50%)	(33)
				SS ratio: 1:10 (g/mL)	
				power: 500 W	
				frequency: 20 kHz	
				time: 10 min	
				yield: 27.1 mg/g	
Green tea	leaves	epigallo catechin gallate	UAE	solvent: ethanol	(34)
				SS ratio: 1:10 g/mL	
				temperature: - 66.53 °C	
				time: 43.75 min	
				yield: 3.86 g/100 g	
Glycyrrhiza uralensis	seed	antioxidant	UAE	Solvent: petroleum ether	(35)
				SS ratio: 1:10 (g/mL)	
				temperature: 50 °C	
				time: 40 min	
				power: 400W	
				yield: 0.129 mg/mL	
Asparagus cultivars	roots	phytochemical compounds	UAE	solvent: petroleum ether	(36)
				SS ratio: 1:40 (g/mL)	
				temperature: 50 °C	
				time: 120 min	
				power: 550 W	
				yield: - 71.1 mg/g	
Camellia oleifera	shell	antioxidant	UAE	solvent: DI H_2_O	(37)
				SS ratio: 10:50 mL/g	
				temperature: 70 °C	
				time: 10 min	
				power: 400 W	
				yield: 12.94 mg/g	
Pumpkin	seed	oil	UAE	solvent: *n*-Hexane (100 mL)	(32)
				SS ratio: 2–6 mL/g	
				time: 34.37 min	
				amplitude: 20–40%	
				yield: 447.4 mg/100g	
Basil	leaves	eugenol	UAE	solvent: ethanol	(31)
				SS ratio: 10:1 mL/g	
				time: 7 - 12 min	
				amplitude: 70–90%	
				yield: 10.25 mg/g	
Sargassum fusiforme	algae	fucoxanthin	UAE	solvent: methanol	(30)
				SS ratio: 40 mL/g (v/w)	
				time: 27 min	
				temperature: 75 °C	
				power: 500 W	
				frequency: 20 kHz	
				amplitude: 53%	
				yield: 34.31 mg/g	
Acer truncatum	seed	oil	SSE	SS ratio: 1:2 (w/v)	(42)
				solvent: butane	
				temperature: 52 °C	
				time: 44 min	
				enthalpy: 20.06 kJ/mol	
				entropy: 70.02 J/(mol. K)	
				yield: 94.50% (v/v)	
Hermetia illucens	larvae	oil	SSE	SS ratio: 3:4 (w/v)	(44)
				solvent: water	
				temperature: 25–65 °C	
				time: 20–60 min	
				solvent: butane	
				yield: 31.19% (w/w)	
Flaveria bidentis	flower	sulfated flavonoids	SSE	SS ratio: 3:30 (w/v)	(47)
				solvent: water	
				temperature: 101.2 °C	
				flowtime: 2.86 mL/min	
				yield: 39.43 mg/100g	
Crude palm oil	fruit	β-carotene	SSE	SS ratio: 1:5 (w/v)	(48)
				solvent: water	
				temperature: 160 °C	
				time: 60 min	
				pressure: 40 bar	
				yield: 857.72 ppm	
Apple pomace	fruit	pectin	SSE	SS ratio: 1:10 (w/v)	(49)
				solvent: water	
				temperature: 140 °C	
				time: 5 - 15 min	
				pressure: bar	
				yield: 35.5 μg/g	
Passiflora edulis	fruit	pectin	SSE	SS ratio: 14.61(w/w)	(50)
				solvent: water	
				temperature: 100–160 °C	
				flowtime: 10g/min	
				yield: 27.6%	
Ganoderma lucidum (G. lucidum) and barley	grain	β-glucan	SSE	SS ratio: 1:50(w/w)	(51)
				temperature: 100 - 190 °C	
				time: 180 min	
				pressure: 1–10 MPa	
				yield: 96.1%	
Edible seaweeds	plant	multiclass contaminants	SPME	solvent: ethanol: acetone	(52)
				SS ratio: 0.25/8 (w/v) (g/mL)	
				temperature: 60 °C	
				time: 55 min	
				yield: 30 μg/kg	
Polymeric ionic liquid		unsaturated compounds	SPME	solvent: acetonitrile	(54)
				SS ratio: 1:2 (v/v)	
				temperature: 35 °C	
				time: 2 h	
				yield: 109 μg/L	
Orange, peach, apple, etc.	fruit	thiabendazole	SPME	solvent: DES	(57)
				SS ratio: 5:600 (v/v) (ml/μL)	
				temperature: 40 °C	
				time: 10 min, 4000 rpm	
				yield: 0.4–150 μg/L	
Atrazine		organic pollutants	SPME	solvent: chloroform: toluene	(53)
				SS ratio: 1:1 (v/v) (mL/μL)	
				temperature: 250 °C	
				time: 15 min	
				yield: 0.063 ng/mL	
Penicillium	fungi	volatile metabolites	SPME	solvent: silica fiber	(60)
				temperature: 25 °C	
				time: 30 min	

#### 2.1.2 Advantages of MAE

MAE can significantly reduce extraction times compared to traditional methods. The application of microwaves allows for rapid heating of the solvent and sample, leading to faster extraction kinetics that can result in higher yields of bioactive compounds compared to conventional extraction methods.^8,9^ The use of microwaves often allows for extraction at lower temperatures, reducing the need for large amounts of solvents. This can contribute to the method’s environmental friendliness and decrease the overall cost of the extraction process.^10^ Microwaves can selectively target specific compounds based on their polarities and dielectric properties. This selectivity is advantageous for extracting target bioactive components while minimizing the extraction of unwanted compounds.^10^ The ability of MAE to control and limit the temperature during extraction helps in preserving thermally sensitive bioactive compounds that may degrade at higher temperatures.^13^ MAE is a relatively simple and straightforward extraction technique. It eliminates the need for complex setups and extended extraction times, making it convenient for laboratory and industrial applications.^10^ As MAE often requires lower temperatures and shorter extraction times, it can contribute to a reduction in energy consumption and the overall environmental impact of the extraction process.^15^ MAE can be applied to a wide range of sample types, including plant materials, foods, and natural products. Its versatility makes it suitable for various research and industrial applications.

#### 2.1.3 Limitations of MAE

Microwave energy has a limited penetration depth into the sample. This can lead to uneven heating, especially in complex matrices, resulting in incomplete extraction of bioactive compounds.^14^ If not carefully controlled, the use of microwaves can lead to localized overheating of certain areas in the sample, causing degradation or alteration of heat-sensitive compounds.^14^ While MAE can offer selective extraction based on the dielectric properties of compounds, achieving high selectivity can be challenging for complex samples with diverse chemical compositions.^14^ Microwave extraction equipment can be relatively expensive, and maintenance costs may be higher compared to some traditional extraction methods.^15^ The use of microwaves raises safety concerns, particularly regarding potential exposure to electromagnetic radiation. Safety precautions and appropriate shielding are necessary to protect operators and researchers.^16^ The interaction of microwaves with matrix components can sometimes lead to the release of interfering substances, affecting the purity of the extracted bioactive compounds.^16^

Despite these limitations, MAE remains a valuable extraction technique, particularly for heat-stable compounds and when time efficiency is crucial. Researchers continue to explore ways to overcome these limitations and enhance the applicability and effectiveness of microwave-assisted extraction in various fields of study.^17^ Overall, microwave-assisted extraction is a powerful and efficient extraction technique that has gained popularity due to its ability to accelerate the extraction process, improve yields, and enhance the extraction of thermally sensitive compounds. Ongoing research continues to explore and refine this technique for various applications.

#### 2.1.4 Recent Advances in MAE

Deep eutectic solvents (DES) have become integral in MAE for extracting bioactive compounds, offering a sustainable and efficient alternative. Comprising safe constituents, DES demonstrates low toxicity and high solubilizing capacity. In the study by Turan et al.,^12^ extraction of pectin from orange peels using MAE, a choline chloride: formic acid DES mixture at 8% (v/v) plays a crucial role. This specific DES enhances pectin extraction significantly, yielding an impressive 46.09% (w/w), which was a noteworthy improvement over conventional method. Microwave-assisted DES extraction notably reduces extraction time by 75%, from 60 to 15 min.^12^ The obtained pectin samples exhibit high methoxyl content, suitable for diverse applications. The study underscores DES as an effective, sustainable, and eco-friendly alternative, emphasizing its potential to revolutionize pectin extraction from natural sources. This aligns with the global push for environmentally conscious practices in industries such as food and pharmaceuticals.^12^ The numerical values, including the substantial pectin yield of 46.09% (w/w) and the impressive 75% reduction in extraction time, highlight the practical significance of DES in enhancing extraction efficiency and promoting eco-friendly methodologies in bioactive compound extraction processes.^12^

The integration of combined microwave extraction is indispensable for maximizing the extraction of bioactive components from natural sources, aligning with the demand for sustainable and time-efficient processes in industries like pharmaceuticals, food, and cosmetics. Combined microwave extraction utilizes the synergistic effects of microwave energy and other extraction mechanisms like solvents, ultrasound, mechanical agitation etc. by coupling microwave radiation with these techniques. This innovative approach synergizes the rapid heating capabilities of microwaves with other methods, such as ultrasound, accelerating the extraction process, reducing times, and enhancing bioactive compound yields. The study by Zhang et al.^13^ investigates the impact of combined ultrasonic microwave-assisted extraction on dictyophora indusiate polysaccharides, revealing that ultrasonic-microwave-assisted extraction (UAME) induces a higher degree of cell wall damage, elevating overall antioxidant capacity. Despite similar chemical composition across methods, UAME excels with a polysaccharide yield of 12.66% (w/w). UAME-extracted polysaccharides exhibit significantly heightened antioxidant activities, with a maximum scavenging rate of 83.23%, surpassing other methods. This combined microwave-ultrasonic approach not only optimizes extraction efficiency but also produces polysaccharides with superior antioxidant properties, showcasing its potential for advanced functional food applications.

### 2.2 Supercritical Fluid Extraction (SFE)

Supercritical fluid extraction (SFE) is a technique used to extract desired compounds from a variety of materials using supercritical fluids as the solvent. A supercritical fluid refers to a substance that is at a temperature and pressure above its critical point, where it exhibits properties of both a liquid and a gas.^17^

**Figure 2:**
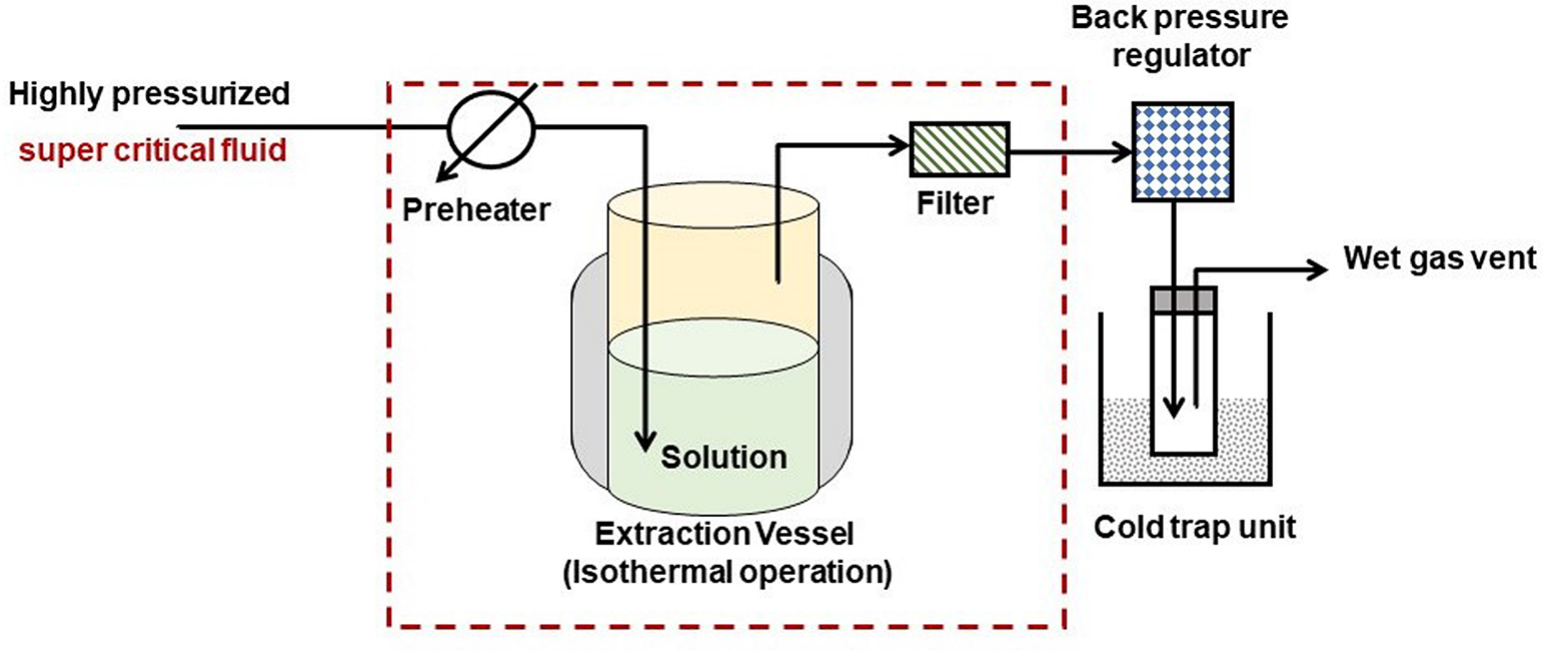
Diagram of supercritical fluid extraction.

A schematic diagram of the extraction process is as shown in the Figure 2. In SFE, the most commonly used supercritical fluid is carbon dioxide (CO_2_) due to its favorable properties, such as low toxicity, availability, and relatively low critical point. However, other supercritical fluids like ethane, propane, and water can also be used depending on the application. SFE involves the use of a specialized extraction system.^17^ The extraction vessel is a high-pressure chamber where the sample to be extracted is placed. It is typically made of stainless steel and equipped with temperature and pressure controls. A high-pressure storage vessel contains supercritical fluid, usually carbon dioxide (CO_2_), which is the most commonly used solvent in SFE. The fluid is pressurized using a pump and heated to reach its supercritical state. A pump is used to transfer the supercritical fluid from the storage vessel to the extraction vessel. The flow rate and pressure of the fluid are carefully controlled to optimize extraction efficiency. Before entering the extraction vessel, the supercritical fluid passes through a preheater to ensure that it reaches the desired temperature for extraction. The preheater is typically a coiled tube immersed in a heating bath. The supercritical fluid is introduced into the extraction vessel, where it comes into contact with the sample. The fluid penetrates the sample matrix, dissolving and extracting the target compounds. The duration of the extraction can vary depending on the nature of the sample and the desired compounds. In some cases, a cosolvent may be added to enhance the extraction of certain compounds. The cosolvent can be introduced at a specific point in the process, either before or during the extraction, depending on the requirements. After passing through the sample, the supercritical fluid, now laden with the extracted compounds, enters a separator vessel. In the separator, the pressure is reduced, causing the supercritical fluid to transition back to its gaseous state. This process is known as the expansion or depressurization step. The extracted compounds, now in gaseous form or as a solute in a liquid cosolvent, are collected in a separate vessel. If a liquid cosolvent was used, then it may be evaporated to obtain the pure extracted compounds.^18^ The supercritical fluid, which has now returned to its gaseous state, can be recycled and reintroduced into the system for further extractions. Alternatively, it may be safely vented or subjected to further purification processes, depending on environmental regulations and the specific requirements of the extraction. It is important to note that the actual setup and components of an SFE system can vary depending on factors such as the scale of the extraction, the nature of the sample, and the desired compounds.^19^

#### 2.2.1 Key Parameters for Efficiency and Yield Enhancement in SFE

##### 2.2.1.1 Pressure

Pressure plays a vital role in SFE as it directly impacts the solvation power and density of the supercritical fluid. By adjusting pressure, the fluid can transition between subcritical and supercritical states, impacting its ability to penetrate and extract compounds from raw materials. In the supercritical fluid extraction of triglycerides from milk thistle seeds, as studied by Palaric et al.,^18^ pressure significantly influenced extraction efficiency. The highest extraction of triglycerides (20% w/w) was achieved with pure CO_2_ at 25 MPa, 40 °C, in 30 min, demonstrating that pressure is a critical parameter in the supercritical fluid extraction process, yielding the highest fluid density, crucial for solvation power. Extraction efficiency notably increased with higher pressure, while the impact of temperature was less distinct. At 15 MPa, temperature slightly favored extraction, and at 25 MPa, it remained relatively constant. This behavior suggests that solvation power is not solely dependent on fluid density but may involve enhanced compound diffusion at higher pressures. In another similar work, Almeida et al.,^19^ reported the recovery of ergosterol from agaricus bisporus mushrooms via SFE, and studied the effect of pressure on extraction parameters. At a pressure of 100 bar, the extraction yield and ergosterol recovery were found to be 1.090% and 5.97 ± 0.03 mg ergosterol/g. However, the higher recovery was obtained as 6.23 mg ergosterol/g, at 244 bar. Higher pressures enhance solubility, promoting efficient extraction of target components. Controlling pressure allows for the customization of extraction conditions based on the nature of the compounds of interest. Additionally, pressure influences mass transfer kinetics, affecting the extraction rate.^19^ Optimal pressure selection is vital for maximizing extraction efficiency, ensuring selective compound recovery, and minimizing environmental impact, making pressure a key parameter in the optimization of SFE processes.

##### 2.2.1.2 Temperature

Temperature is a critical factor in SFE as it manipulates the fluid’s density, diffusivity, and overall solvation power. Temperature control allows the adjustment of the fluid’s critical parameters, impacting its ability to dissolve specific compounds.^20,21^ In the study by Hu et al.,^20^ the effect of temperature on the extraction of flavonoids from dendrobium chrysotoxum flowers using SFE was investigated. The optimal conditions for SFE were determined as extraction time of 90 min, temperature of 50 °C, and pressure of 20 MPa. The total flavonoid extraction yield under these conditions was 2.04% ± 0.02% (g/g). The SFE temperature range of 40 to 50 °C resulted in a higher yield, reaching its peak at 50 °C. Overall, higher temperatures were associated with increased flavonoid extraction efficiency, as indicated by the total flavonoid extraction yield values.^20^ Palaric et al.^18^ investigated the effect of temperature on the SFE of lipids and flavonolignans from milk thistle seeds using carbon dioxide as the solvent. For lipid extraction, temperatures of 40 and 60 °C were considered at pressures of 15 and 25 MPa. Results showed that at 40 °C and 25 MPa, the optimal conditions for lipid extraction, the fluid density was 0.88 g/mL. Increasing pressure from 15 to 25 MPa enhanced extraction, while the impact of temperature is less distinct. For flavonolignans, extraction at 40 °C and 25 MPa using a modifier (20% ethanol) showed improved recovery.^18^ Higher temperatures generally increase extraction efficiency by enhancing mass transfer kinetics, promoting the diffusion of solutes into the supercritical fluid. However, the temperature must be carefully optimized to avoid thermal degradation of sensitive compounds. Temperature control is essential for achieving selectivity in extraction processes, tailoring SFE for various applications, from pharmaceuticals to natural product extraction. The significance of temperature lies in its role as a versatile tool to optimize extraction conditions for optimal and selective compound recovery in SFE.

##### 2.2.1.3 Solid to Solvent Ratio

The solid-to-solvent ratio is a crucial parameter in SFE with substantial importance and significance. The effectiveness and productivity of the extraction procedure are directly impacted by this ratio. The study by Almeida et al.^19^ aimed to optimize ergosterol recovery from agaricus bisporus mushrooms through SFE using response surface methodology. The solvent-to-mushroom ratio significantly influenced the extraction yield, ergosterol purity, and ergosterol recovery. The results demonstrated a linear relationship between extraction yield and solvent to mushroom ratio, indicating that the limiting step in extraction was compound solubility rather than mass transfer limitations. Ergosterol recovery increased up to an solvent to mushroom ratio of 300 mL/g, reaching a plateau at approximately 6.2 mg/g. The study identified an optimal compromise at a solvent to mushroom ratio of 300 mL/g, maximizing extraction yield and ergosterol recovery while compromising purity.^19^ Similarly, in the study by Hu et al.,^20^ the effect of solid-to-solvent ratio on the extraction of flavonoids from dendrobium chrysotoxum flowers was investigated using SFE.^20^ The solid-to-solvent ratio, expressed as 1:40 g/mL (w/v), was found to be optimal for SFE. The results showed that at optimal conditions of SFE were time 90 min, temperature 50 °C, pressure 20 MPa, the total flavonoid extraction yield was determined to be 2.04% ± 0.02% (w/w). The specified solid-to-solvent ratio of 1:40 played a crucial role in achieving the maximum flavonoid extraction yield using SFE, contributing to the economic viability of the process.^20^ Proper optimization of the solid-to-solvent ratio ensures the extraction of target compounds while maintaining economic feasibility. An insufficient ratio may lead to incomplete extraction, wasting the potential yield of valuable compounds from the solid matrix. Alternatively, an excessive ratio may not only be economically impractical but can also result in decreased mass transfer and reduced extraction efficiency.^20^ Achieving an optimal balance is crucial for maximizing the concentration of desired compounds in the extract while minimizing the consumption of supercritical fluid, contributing to the cost-effectiveness and sustainability of the SFE process. Consequently, understanding and controlling the solid-to-solvent ratio play a pivotal role in the successful application of supercritical fluid extraction across various industries, including pharmaceuticals, food, and natural product extraction.

##### 2.2.1.4 Extraction Time

The extraction time in SFE is of paramount significance as it determines the cost and feasibility of the extraction process. The duration of SFE determines the interaction time between the supercritical fluid and the target compounds in the sample. The study by Hasanov et al.,^22^ SFE of flaxseed oil at different time scales revealed significant economic implications. In the 1 h extraction scenario, the 500 L SFE system demonstrated a feasible techno-economic profile with a low manufacturing cost of 5.45 US $/kg extract and a short payback time of 3.55 years, considering a selling price of 10 US $/kg oil and a raw material cost of 0.83 US $/kg. However, extending the extraction time to 2 h increased the cost of manufacturing and payback time. The maximum oil recovery was 81% at 60 min and 90.6% at 120 min. The study emphasized the critical role of extraction time, where shorter batch times resulted in higher biomass and oil production, leading to lower cost of manufacturing. Overall, the findings underscored the economic viability of SFE for flaxseed oil production, especially at higher scales and shorter extraction times. Similarly, the study by Frohlich et al.,^23^ compared the efficiency of SFE and pressurized liquid extraction for obtaining clove leaf extracts, focusing on the impact of time on yield, eugenol content, and antioxidant activity. In SFE, the extraction time of 80 min resulted in a highest yield of 1.94 wt %, with an eugenol content of 0.806 wt %. SFE demonstrated higher efficiency for eugenol-rich extracts, emphasizing the importance of time and pressure. Both methods showed satisfactory antioxidant activity. Overall, the study highlighted the influence of extraction time on the yield and composition of clove leaf extracts, providing insights into the optimal conditions for different extraction techniques. However, the study by Alwazeer et al.^24^ employed SFE using CO_2_ to extract phytochemicals from various agri-food wastes. The SFE conditions involved a temperature of 30 °C, pressure of 70 bar, and a duration of 2 h. The comparison was made with hydrogen-rich water extraction and pure water extraction. Results demonstrated that hydrogen-rich water extraction outperformed SFE and pure water extraction in terms of extracting phenolic compounds, flavonoids, anthocyanins, and antioxidants. The percent increase in total phenolic content ranged from 5.14% to 12.06%, and 22.27% to 49.62% for SFE and hydrogen-rich water extraction, respectively. Optimal extraction time ensures thorough solvation of the desired components, maximizing extraction yield. However, prolonged extraction times may lead to diminishing returns or degradation of thermally sensitive compounds. Therefore, understanding and optimizing extraction times in SFE are crucial for achieving high extraction efficiency, maintaining product quality, and minimizing energy consumption. Summary of some of the recent SFE studies reported in the literature are given in Table 1.

##### 2.2.1.5 Solvent

The choice of solvent in SFE impacts the efficiency and selectivity of the extraction process. Supercritical fluids, typically carbon dioxide (CO_2_), serve as solvents in SFE due to their unique properties such as tunable density and polarity near their critical point. The study by Rodrigues et al.^21^ compared the efficiency of SFE with Soxhlet extraction for extracting lupine-triterpenoids from Acacia dealbata Link bark. SFE was conducted using carbon dioxide (CO_2_) with or without cosolvents (ethyl acetate or ethanol) at different pressures, temperatures, and cosolvent contents. The highest total extraction yield achieved was 1.57 wt %, with significant extraction yields and concentrations of lupenyl acetate (LA) and lupenone (Lu). SFE outperformed Soxhlet extraction in terms of productivity and selectivity, particularly under specific conditions. The addition of cosolvents and higher pressure and temperature in SFE favored productivity, reducing the production cost. However, the selectivity of SFE for lupane triterpenoids was impacted by the choice of cosolvent, with pure CO_2_ exhibiting better selectivity. Economic analysis revealed that despite higher investment costs, the addition of ethanol improved productivity, leading to lower overall extraction costs compared to pure CO_2_. Overall, SFE demonstrated superior efficiency and economic viability for extracting lupane-triterpenoids from Acacia dealbata Link bark compared to Soxhlet extraction. However, the study by Alwazeer et al.,^23^ evaluated hydrogen-rich water extraction and SFE for extracting phytochemicals from various agri-food wastes. Hydrogen-rich water extraction consistently outperformed SFE and pure water extraction, yielding higher total phenolic content, total flavonoid content, total anthocyanin, and antioxidant activity (DPPH and ABTS) across all plant wastes. Percentage increases (%) ranged between 5.14 and 12.06 (total phenolic content), 1.32–35.59 (total flavonoid content), 18.18–53.19 (anthocyanins), 2.21–22.37 (DPPH), and 1.16–7.49 (ABTS) for SFE, and 22.27–49.62 (total phenolic content), 16.01–53.03 (total flavonoid content), 80.53–390.1 (anthocyanins), 9.03–142.46 (DPPH), and 14.47–28.05 (ABTS) for hydrogen-rich water extraction. It demonstrated superior extraction efficiency for all phytochemical types compared to SFE, particularly for nonflavonoids such as phenolic acids and flavonoids. The study recommends hydrogen-rich water extraction due to its simplicity, low cost, nontoxic solvent use, environmental friendliness, and higher extraction efficiency compared to SFE. These findings highlight the potential of hydrogen-rich water extraction as a novel extraction method for obtaining phytochemicals from plant sources.

These solvents offer several advantages, including shorter extraction times, minimal usage of organic solvents, higher extraction efficiency, and automation capabilities. However, the effectiveness of SFE can vary based on the solvent’s properties and the target compounds being extracted. Researchers continuously explore novel solvents and solvent mixtures to enhance the performance and versatility of SFE for extracting a wide range of compounds from various matrices.

#### 2.2.2 Advantages of SFE

Supercritical fluids can be perfected to selectively extract specific compounds by adjusting pressure and temperature, providing a high degree of selectivity.^20^ Since supercritical CO_2_ is used as the solvent, there are no residual solvents left in the extracted product, making it suitable for food, pharmaceutical, and other sensitive applications.^20^ SFE is typically carried out at relatively low temperatures compared to other extraction methods, reducing the risk of thermal degradation of heat-sensitive compounds.^20^ SFE is generally faster than traditional extraction methods, leading to higher productivity and efficiency.^21^ CO_2_ is a readily available, nontoxic, nonflammable, and environmentally friendly solvent for SFE. The extracted products often have a high purity level because the supercritical CO_2_ selectively extracts the target compounds without pulling in unwanted impurities.^22^ SFE can be applied to a wide range of compounds, including lipids, essential oils, flavors, fragrances, and bioactive compounds.^22^

#### 2.2.3 Limitations of SFE

The equipment required for supercritical fluid extraction is expensive, which may be a barrier for smaller laboratories or facilities.^22^ Operating SFE equipment requires trained personnel due to the complexity of controlling pressure and temperature conditions.^23^ Supercritical CO_2_ has limited solubility for polar compounds, which may result in lower extraction efficiency for certain types of compounds.^23^ The scale of supercritical fluid extraction is often limited to batch processing, and scaling up to larger volumes may pose challenges.^23^ Supercritical CO_2_ can be sensitive to the presence of moisture, which might affect the extraction efficiency and product quality.^23^ While the process itself is carried out at lower temperatures, the compression of CO_2_ to supercritical conditions can require significant energy input.^24^ Achieving optimal extraction conditions can be challenging, and small variations in parameters may affect the efficiency and selectivity of the extraction process.^23^

#### 2.2.4 Recent Advancements in SFE

In SFE, using a supercritical fluid alone may limit the solubility of certain compounds, but the addition of a cosolvent, such as ethanol, broadens the range of extractable substances. Cosolvents modify the solvation properties of the supercritical fluid, improving the dissolution of target compounds and increasing extraction yields. They influence mass transfer characteristics, affecting external diffusion coefficients and overall extraction rates. Additionally, cosolvents can alter the selectivity of the extraction process, making it adaptable for different applications. In the study on the SFE of carotenoids from microalgae by Sanchez et al.,^25^ the solvent systems used were supercritical CO_2_ alone and CO_2_ with 5% ethanol as a cosolvent. The extraction behavior and yield of carotenoid was controlled by their mass transfer with the cosolvent using different biomass. The cosolvent enhances the solubility of carotenoids in supercritical carbon dioxide, improving the overall extraction efficiency. It was observed that using biomass *Synechococcus* sp., the rate of extraction and the yield were improved by adding 5% ethanol with CO_2_. This indicated that the addition of cosolvent was favorable for the extraction of carotenoid.

Another advancement in SFE is sequential supercritical extraction. This is a multistage process involving successive extraction steps using supercritical fluids, often carbon dioxide, with or without cosolvents like ethanol. The technique provides superior extraction efficiency and selectivity compared to single-stage processes. The study by Marillan et al.,^26^ investigated the extraction of bioactive compounds from Leptocarpha rivularis stems using three-stage sequential supercritical extraction with CO_2_. The experiments were conducted, varying temperature (40–60 °C) and pressure (20–50 MPa). On comparing the process configurations, it was observed that the total yield, i.e., the yields of terpenes (699.9 mg linalool/kg), flavonoids (741.9 mg quercetin/kg), and alkaloids (6.22 mg atropine/kg) obtained in sequential supercritical extraction were greater than the one-stage extraction. This multistage approach allowed better substrate exhaustion and higher yields compared to one-stage extraction, demonstrating the significant improvements achievable in bioactive compound extraction from plant materials.

The application of machine learning in supercritical fluid extraction (SFE) is pivotal for revolutionizing the field. Machine learning accelerates the estimation of critical properties, solubilities, and miscibilities, streamlining the optimization of SFE processes. Additionally, machine learning facilitates the understanding of the inhomogeneous nature of supercritical fluids, providing insights that contribute to the development of efficient and reliable extraction methods. In the reviewed study by Roach et al.,^27^ machine learning is applied to various aspects of supercritical fluids research, including the estimation of thermodynamic properties, solubilities, and miscibilities. For instance, in predicting a supercritical state for H_2_, Cheng et al.,^28^ used machine learning to model potential energy surfaces and interatomic forces, with noteworthy results. This transformative technology holds the potential to enhance the commercial viability of SFE, offering a data-driven approach for rapid advancements in the domain.

### 2.3 Ultrasound-Assisted Extraction

Ultrasound-assisted extraction (UAE) is a technique that employs high-frequency sound waves to enhance the extraction process of target compounds from various samples. UAE utilizes the mechanical vibrations, cavitation, and microstreaming generated by ultrasound waves to increase mass transfer and disrupt cell structures, facilitating the release of the desired compounds.^29,30^ The extraction efficiency in the UAE is influenced by several factors. These include the frequency and intensity of the ultrasound waves, the choice of extraction solvent, extraction time, temperature, etc. These factors are to be carefully adjusted to maximize the extraction efficiency without causing degradation or thermal damage to the target compounds. UAE encompasses various methods, two prominent ones being bath extraction and horn extraction. Bath extraction involves either immersing the sample vessel in a liquid medium (mostly water) or directly putting in the bath exposed to ultrasonic waves.^31^ Horn extraction, however, utilizes ultrasonic horns directly applied to the sample. Both the technologies vibrations create cavitation, breaking down cellular barriers and accelerating the release of desired compounds. Both techniques offer unique advantages, such as reduced processing time and improved yields, making them versatile tools for extracting bioactive components from diverse sources in fields ranging from pharmaceuticals to food processing.^32^ However, they are different in terms of the types of cavities generated and cavitational impurities produced as a result of cavity collapse.

#### 2.3.1 Ultrasonic Bath Extraction

**Figure 3:**
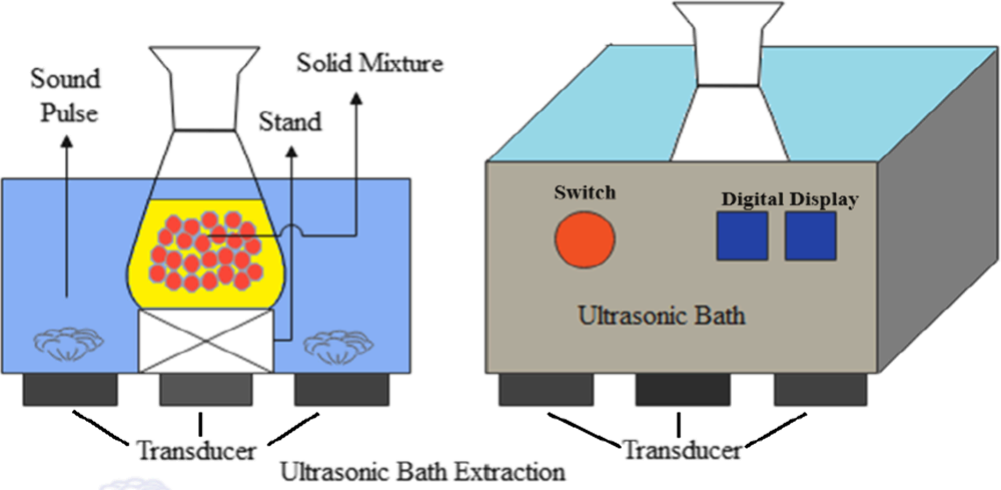
Diagram of an ultrasonic bath.

Ultrasonic bath extraction is a powerful and widely used technique for extracting bioactive compounds from various materials, including plant extracts, pharmaceuticals, and natural products. In this method, the sample is immersed in a liquid solvent within a bath or tank, and ultrasonic waves are applied to the entire solution.^32^ A schematic diagram of the ultrasound bath extraction experimental set is shown in Figure 3.

Ultrasound generator produces high-frequency sound waves typically in the range of 20 kHz to several megahertz, depending on the application. The transducer converts electrical energy from the generator into mechanical vibrations, creating ultrasonic waves. The “Bath” or “Tank” Contains the liquid sample and facilitates even distribution of ultrasound waves throughout the solution. Ultrasonic waves induce cavitation, the formation and collapse of microscopic bubbles. The implosion of these bubbles generates intense local heating and pressure, breaking cell walls and enhancing the release of bioactive compounds.^33^ To prevent excessive heating during the extraction process, a temperature control system may be integrated into the ultrasonic bath. Ultrasonic bath extraction offers advantages such as rapid extraction, increased yields, and reduced solvent consumption. It is particularly effective for heat-sensitive. The method is scalable, making it suitable for both laboratory research and industrial production.

#### 2.3.2 Ultrasonic Horn Extraction

Ultrasonic horn extraction, also known as probe or sonotrode extraction, is a specialized ultrasonic technique employed for the efficient extraction of bioactive compounds from various materials. The extraction process involves a solid metal horn or probe that directly contacts the sample. The horn is designed to transmit ultrasonic vibrations efficiently and is often tapered to amplify the intensity at its tip. The ultrasonic transducers are similar to bath extraction, an ultrasonic transducer generates high-frequency sound waves (typically 20 kHz to several megahertz) that are applied directly to the horn.^32^ The horn’s vibrations induce cavitation at the tip, creating microbubbles in the surrounding liquid. The implosion of these bubbles generates localized pressure and temperature changes, effectively disrupting cell structures and facilitating the extraction of target compounds. The sample is typically placed in a container with a liquid solvent, and the ultrasonic probe is immersed directly into the solution.^33^

**Figure 4:**
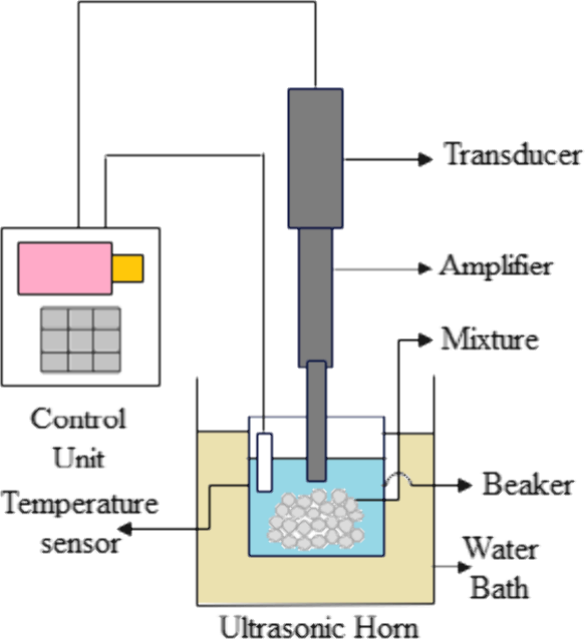
Diagram of the ultrasonic horn.

A schematic diagram of extraction using ultrasonic horn is as shown in Figure 4. The extraction process can be optimized by adjusting parameters such as ultrasonic frequency, power, and extraction time to optimize the yield and quality of the extracted compounds. Ultrasonic horn extraction is particularly useful for tough and fibrous materials where traditional methods may be less efficient. It finds applications in extracting bioactive compounds from plant tissues, herbal products, and various natural sources.^34^ This method offers advantages such as rapid extraction, increased yields, and enhanced selectivity. It is also valuable for its ability to operate at lower temperatures, preserving heat-sensitive compounds. Ultrasonic horn extraction is employed in research laboratories and industrial settings, especially in the pharmaceutical, nutraceutical, and food industries, where precise control over extraction parameters is crucial for obtaining high-quality extracts.^34^

#### 2.3.3 Cavitation Phenomenon

**Figure 5:**
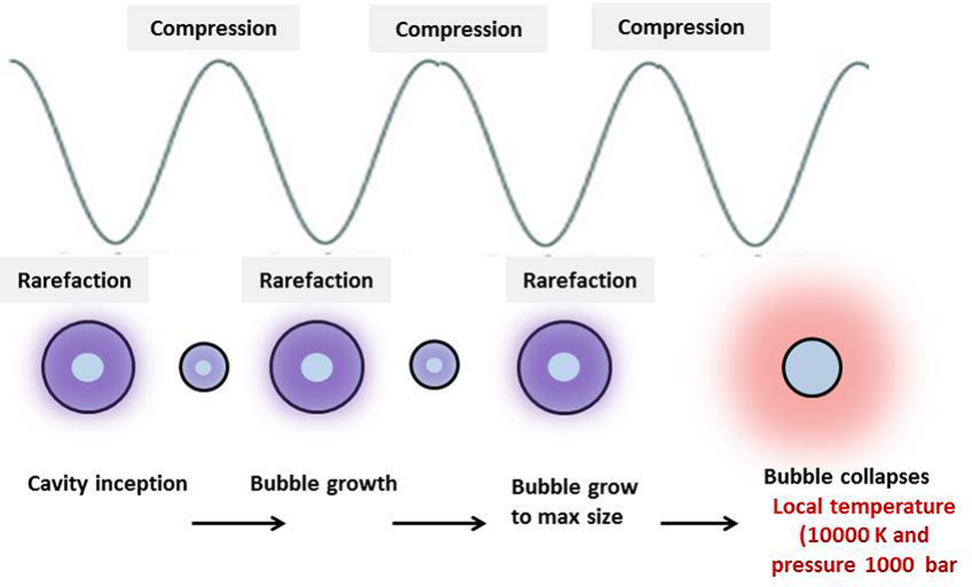
Cavitation phenomenon in UAE.

Cavitation in UAE is the formation and subsequent collapse of microscopic bubbles in a liquid medium due to ultrasonic waves. Cavitation in UAE is the formation and subsequent collapse of microscopic bubbles in a liquid medium due to ultrasonic waves. A schematic diagram of cavitation is as shown in Figure 5. When ultrasonic waves pass through a liquid, they create alternating high and low-pressure zones.

During the low-pressure phase, small gas or vapor bubbles are form. As these bubbles grow and become unstable, they collapse violently during the high-pressure phase. This rapid bubble collapse generates localized hot spots with extreme temperatures and pressures, leading to intense microstreaming and turbulence in the liquid. In UAE, this phenomenon enhances mass transfer between the solid material (e.g., plant cells) and the solvent, accelerating the extraction of bioactive compounds. This cavitation-driven agitation improves extraction efficiency, reduces extraction times, and often allows for milder operating conditions, making UAE a powerful extraction technique.

#### 2.3.4 Key Parameters for Efficiency and Yield Enhancement in UAE

##### 2.3.4.1 Ultrasonic Power Amplitude

Ultrasonic power amplitude plays a crucial role in UAE of bioactive compounds. The amplitude determines the intensity of ultrasonic waves, influencing cavitation, a phenomenon crucial for breaking cell walls and enhancing mass transfer. Nie et al.^30^ reported the UAE of fucoxanthin from sargassum fusiforme using green solvents. In the study, the effect of ultrasonic power amplitude on the yield of fucoxanthin from *Sargassum fusiforme* was examined using ethyl lactate as the solvent. The power amplitude was varied from 20 to 70% and the optimal amplitude was identified as 50%, at which the yield of fucoxanthin was maximum. At higher amplitudes (>50%), the yield was not significantly increased due to the potential degradation of fucoxanthin at excessive energy dissipation. Thus, an amplitude of 50% provided the best balance between maximizing yield and maintaining process efficiency. The study concludes that fine-tuning the ultrasonic amplitude is crucial for optimizing the extraction process and achieving high yields of fucoxanthin using green solvents. In the optimization study of UAE of eugenol-rich fraction from *O. basilicum* by Kousar et al.,^31^ the amplitude significantly influenced various responses. The optimal conditions obtained through numerical optimization were solvent concentration (52.48%), amplitude (90%), and sonication time (7.75 min). The extract yield ranged from 55.48 wt % (at 70% amplitude) to 80.4 wt % (at 90% amplitude), and the model indicated a positive effect of amplitude and a negative effect of sonication time on yield. The eugenol content, a major compound, was found to be 41.44 wt %. The negative coefficient of sonication time suggested a decrease in yield with increased sonication time. The study demonstrated the successful application of response surface methodology for the optimization of UAE conditions, highlighting the significance of amplitude in the extraction process. Similarly, in the study by Singh et al.,^32^ the effect of amplitude in UAE of pumpkin seed oil was investigated. The amplitude was varied in the range of 20–40%, and its impact on oil yield, total phenolic content, squalene content, and oil induction time was analyzed. The results indicated that the oil yield was increased until an amplitude of 35% and thereafter remain unchanged. Additionally, amplitude showed a quadratic effect on total phenolic content and squalene content, with an optimal amplitude of 35%, where total phenolic content and squalene content exhibited a peak. The optimal amplitude for UAE was determined to be 35%, resulting in improved oil yield (39.05%), total phenolic content (45.02 mg/g), squalene content (447.4 mg/100g), and oil induction time (5.27 h). Controlling ultrasonic amplitude is necessary not only to maximize extraction efficiency but to avoid its potential adverse impacts on properties of active molecules. Excessive amplitude leads to undesired effects such as increased temperatures and degradation of heat sensitive molecules.^32^

##### 2.3.4.2 Extraction Time

In UAE, extraction time is a critical parameter influencing the efficiency of bioactive compound recovery. The duration of exposure to ultrasonic waves directly impacts the disruption of plant cells and the release of valuable compounds, such as polyphenols. In the study by Christou et al.,^33^ on the UAE of polyphenols from carob pulp, the effect of extraction time on total phenolic content was investigated. UAE of phenolics was carried out using a 500 W power and 20 kHz frequency ultrasonic probe at different sonication time ranging from 5 to 35 min. For continuous ultrasound-assisted extraction, the optimal extraction time was found to be 10 min, whereas for pulsed ultrasound-assisted extraction, the optimal time extended to 14 min. Prolonging the extraction time beyond these optimal durations resulted in a decreased phenolic content due to several factors. Extended sonication increases the likelihood of heat generation, which can degrade the phenolic compounds, reducing their recovery. Additionally, prolonged exposure to ultrasound may lead to the oxidation of bioactive substances, further decreasing the total phenolic yield. The study observed that the total phenolic content was higher with optimal extraction times, achieving values of 121.53 ± 0.82 mg GAE/g carob pulp comparatively. Similarly, in the study by Ayyizdil et al.,^34^ the extraction time significantly influences epigallocatechin gallate yield in conventional hot water and UAE from green tea. In conventional hot water extraction, at 66.53 °C, the optimal time of 43.75 min led to maximum epigallocatechin gallate yield. Similarly, in UAE with ethanol, longer extraction times resulted in higher epigallocatechin gallate extraction rates, with the optimal time being 43.75 min. UAE with ethanol exhibited nearly 100% more epigallocatechin gallate content at optimum conditions compared to conventional hot water extraction. This increase can be attributed to enhanced mass transfer and cell wall disruption facilitated by longer extraction durations. The study highlights the crucial role of extraction time in maximizing epigallocatechin gallate yield (0.39 g/g), underscoring its significance in industrial-scale processes for green tea extraction. The optimized conditions for UAE of epigallocatechin gallate with ethanol were 66.53 °C, 43.75 min, and 67.81% ethanol, demonstrating the importance of precise time control in achieving optimal extraction efficiency.

An optimal extraction time ensures sufficient contact between the solvent and plant matrix, facilitating enhanced mass transfer of bioactive constituents. However, prolonged extraction times may lead to degradation and reduced yields due to factors like heat generation and oxidation. Therefore, precise control of extraction time in the UAE is crucial for maximizing extraction efficiency and maintaining the quality of recovered compounds.

##### 2.3.4.4 Temperature

Temperature is a critical factor in UAE, influencing the efficiency of extracting bioactive compounds from various sources. In this study by Bhadange et al.,^38^ the impact of temperature on d-galacturonic acid extraction from basil seeds was investigated using stirred reactor extraction, Soxhlet extraction, and ultrasonic extraction techniques. The results demonstrated a substantial influence of temperature on the yield of d-galacturonic acid, with ultrasonic extraction exhibiting superior performance. The highest yield of d-galacturonic acid (104.25 mg/g of basil seeds) was achieved at an optimized temperature of 70 °C using ultrasound-assisted extraction, surpassing stirred reactor (92.64 mg/g) and Soxhlet extraction (52.2 mg/g).^38^ The findings underscore ultrasonic extraction as the optimal approach for obtaining higher d-galacturonic acid yields from basil seeds, offering a rapid and energy-efficient alternative to traditional extraction methods.^38^ Temperature significantly influences the extraction of polyphenols, particularly epigallocatechin gallate, from green tea in the conducted study by Ayyildiz et al.,.^34^ Optimal conditions for conventional hot water extraction and UAE with water and ethanol were determined through response surface methodology. The study revealed that high-temperature with longer process time and operations for short duration were ineffective in obtaining sufficient epigallocatechin gallate. The epimerization reactions of epigallocatechin gallate were observed at high temperatures, contributing to lower concentrations in the extracts. For conventional hot water extraction, the optimal temperature, time, and tea-to-water ratio were 87.13 °C, 26.11 min, and 25.71 mL/g, respectively. In UAE with water, the optimal conditions were 79.63 °C and 52.49 min. Notably, UAE with ethanol demonstrated superior efficiency at lower temperatures, with optimal conditions at 66.53 °C, 43.75 min, and 67.81% ethanol, yielding almost 100% more epigallocatechin gallate content compared to conventional methods. The study underscores the importance of temperature control in optimizing green tea extraction processes for enhanced polyphenol yield. Higher temperatures generally increase solubility, promoting the release of target compounds. However, the impact of temperature is a delicate balance, as excessive heat can diminish cavitation intensity. The optimal temperature range varies for different applications, and understanding this interplay is essential for maximizing yields and maintaining the effectiveness of UAE processes in diverse fields such as food, pharmaceuticals, and environmental analysis.

##### 2.3.4.5 Solid to Solvent Ratio

The solid-to-solvent ratio is crucial in UAE as it determines the concentration of bioactive compounds extracted from solid materials. An optimal ratio ensures efficient contact between the material and solvent, enhancing mass transfer and compound solubility. In the UAE of glycyrrhiza uralensis seed protein by Simayi et al.,^35^ the solid-to-solvent ratio played an essential role in persuading the content and yield of seed protein. The solid-to-solvent ratio significantly impacts the yield of Glycyrrhiza uralensis seed protein (GSP-U) during UAE. The solid to solvent ratio was varied in the range of 1:20 to 1:30 (g/mL) and optimized using response surface methodology. It was observed that at an optimum ratio of 1:29, the maximum yield of 15.14% and a GSP-U content of 39.72% was attained. Typically, ultrasound aids in reducing solvent consumption due to enhanced mass transfer, which allows for efficient extraction even at lower solid-to-solvent ratios. In this study, the optimized conditions indicate a relatively low solid-to-solvent ratio, reflecting the efficiency of UAE in extracting GSP-U. Lower solvent consumption not only aligns with sustainable practices but also contributes to cost-effectiveness in large-scale extraction processes.

Similarly, the study by Zhou et al.,^37^ investigated the impact of the solid-to-solvent ratio on the extraction yield of polysaccharides from camellia oleifera fruit shells using UAE. The optimization process, conducted through response surface methodology (RSM), explored various ratios (10, 20, 30 mL/g) to evaluate their influence on polysaccharide yield. Results revealed that the extraction rate exhibited no significant change between 10 and 20 mL/g, reaching its highest (12.94 ± 0.10%) at a ratio of 30 mL/g.^37^ This finding indicates that an optimal balance was achieved, as excess solvent could lead to unnecessary waste and prolonged evaporation concentration time. In UAE, tweaking the amount of solid material compared to the liquid solvent helps boost how well we extract stuff, like important compounds. This adjustment affects how fast we can extract these goodies, letting us customize the process for different materials and use our resources better. So, getting this ratio right is super important for making UAE work really well and making sure we’re doing it in an eco-friendly way. Further, an overview of some of recent UAE studies reported in the literature are explained in Table 1.

#### 2.3.5 Advantages of UAE

Ultrasound enhances mass transfer, breaking down cell walls and membranes, leading to improved extraction efficiency of bioactive compounds from various materials.^30^ Ultrasound accelerates the extraction process, significantly reducing the time required compared to traditional methods, making it a more time-efficient technique.^30^ The cavitation effect created by ultrasound promotes the release of intracellular components, resulting in higher yields of target compounds.^32^ Ultrasound can be tuned to specific frequencies, allowing for selective extraction of certain compounds while preserving others, making it a versatile method for various applications.^32^ The enhanced mass transfer and extraction efficiency often allow for the use of lower solvent volumes, contributing to sustainability and cost-effectiveness.^33^ Ultrasound can operate at lower temperatures compared to traditional methods, minimizing the thermal degradation of heat-sensitive compounds.^33^

#### 2.3.6 Limitations of UAE

Ultrasonic equipment can be relatively expensive to purchase and maintain, particularly at larger scales, which may pose a barrier for some laboratories or small-scale operations.^34^ Ultrasonic extraction requires energy to produce high-frequency waves, and while the process is efficient, energy consumption can be a consideration in large-scale applications.^34^ Proper design of extraction equipment is critical to ensure uniform distribution of ultrasound waves, and this can be challenging for certain materials or sample types.^35^ Overly intense ultrasound conditions may lead to sample degradation, particularly for delicate or heat-sensitive compounds, necessitating careful control of parameters.^36^ Ultrasonic extraction generates noise, and in industrial settings, noise pollution may be a consideration.^37^

In summary, UAE offers numerous advantages in terms of efficiency, selectivity, and reduced processing time, but careful consideration of equipment costs, optimization challenges, and potential sample degradation is necessary for its successful implementation.

#### 2.3.7 Recent Advancements in UAE

The incorporation of hybrid extraction techniques into UAE is of paramount significance in advancing the extraction efficiency of bioactive compounds. Hybrid methods, combining UAE with technologies like MAE, thermal solvent extraction, etc., synergistically exploit different mechanisms, resulting in improved extraction yields and selectivity. The study by Garcia-Ortiz et al.,^39^ highlights the significance of employing hybrid extraction techniques, combining UAE with MAE, for obtaining pigments from differently pigmented corn varieties. The hybrid approach demonstrated superior extraction yields, up to 25% higher than individual techniques. Notably, the content of hydrolyzable polyphenols in the hybrid approach was 15.5% higher than MAE and 77.3% higher than UAE. The concentration of condensed tannins in the combined approach surpassed individual techniques of MAE and UAE by 43.6% (w/w) and 62.8%, respectively. Furthermore, the hybrid technique allowed for the identification of flavonoids like apigenin derivatives. The study underscores the effectiveness of the hybrid technique in enhancing extraction efficiency and obtaining pigments with valuable antioxidant properties from red corn varieties.^39^ The utilization of hybrid extraction techniques by Ozsefil et al.,^40^ combining UAE with thermal methods, is significant for enhancing polyphenol recovery from waste tea biomass. The study demonstrates that waste tea, particularly second sieving waste, exhibits a polyphenol extraction rate exceeding 80%, surpassing tea leaves. The hybrid operation, involving 20 kHz ultrasound and heating at 80 °C, achieves the highest extraction efficiency at 92%. A recovery rate of 84% for ultrasonic extraction at 20 kHz and power intensities of 0.22 Wm/L, 0.023 Wm/L, and 0.068 Wm/L for 20 kHz, 35 kHz, and 200 kHz, respectively, are among the specific figures provided in the results. These figures highlight the quantifiable benefits of using these techniques to efficiently and ecologically safely extract polyphenols.^40^ The utilization of hybrid techniques facilitates precise parameter optimization, allowing researchers to tailor extraction conditions for maximal efficiency.

Recent advancements in ultrasound-assisted extraction have focused on utilizing green solvents, such as ethanol or water, to improve extraction efficiency while reducing environmental impact. These eco-friendly solvents enable enhanced extraction of bioactive compounds from plant materials, offering a sustainable approach to phytochemical extraction with a reduced environmental footprint. In the study of green extraction of bioactive compounds from thuja orientalis by Imtiaz et al.,^41^ use of green solvents (hydro-ethanol) in UAE, plays a crucial role in achieving efficient and environmentally friendly large-scale extraction of bio actives as compare to other advanced techniques. Under optimized conditions using 70% hydro-ethanol, ultrasound-assisted extraction (UAE) yielded notable results: total phenolic content reached 190.5 mg/g, flavonoid content reached 48.59 mg/g, and radical scavenging activity reached 81.73%. UAE. Ongoing UAE advancements, including automation and scale-up capabilities, promise a greener and more efficient alternative to traditional methods, especially for flavonoids and phenolics extraction using 70% hydro-ethanol. This underscores the importance of green solvents in UAE, minimizing solvent and energy consumption while maximizing the extraction of valuable phytochemicals, aligning with sustainability goals in industrial processes. The findings strongly support the use of ultrasound-assisted extraction (UAE) with green solvents as an efficient method for extracting bioactive compounds from natural sources on a larger scale.

### 2.4 Subcritical Solvent Extraction

**Figure 6:**
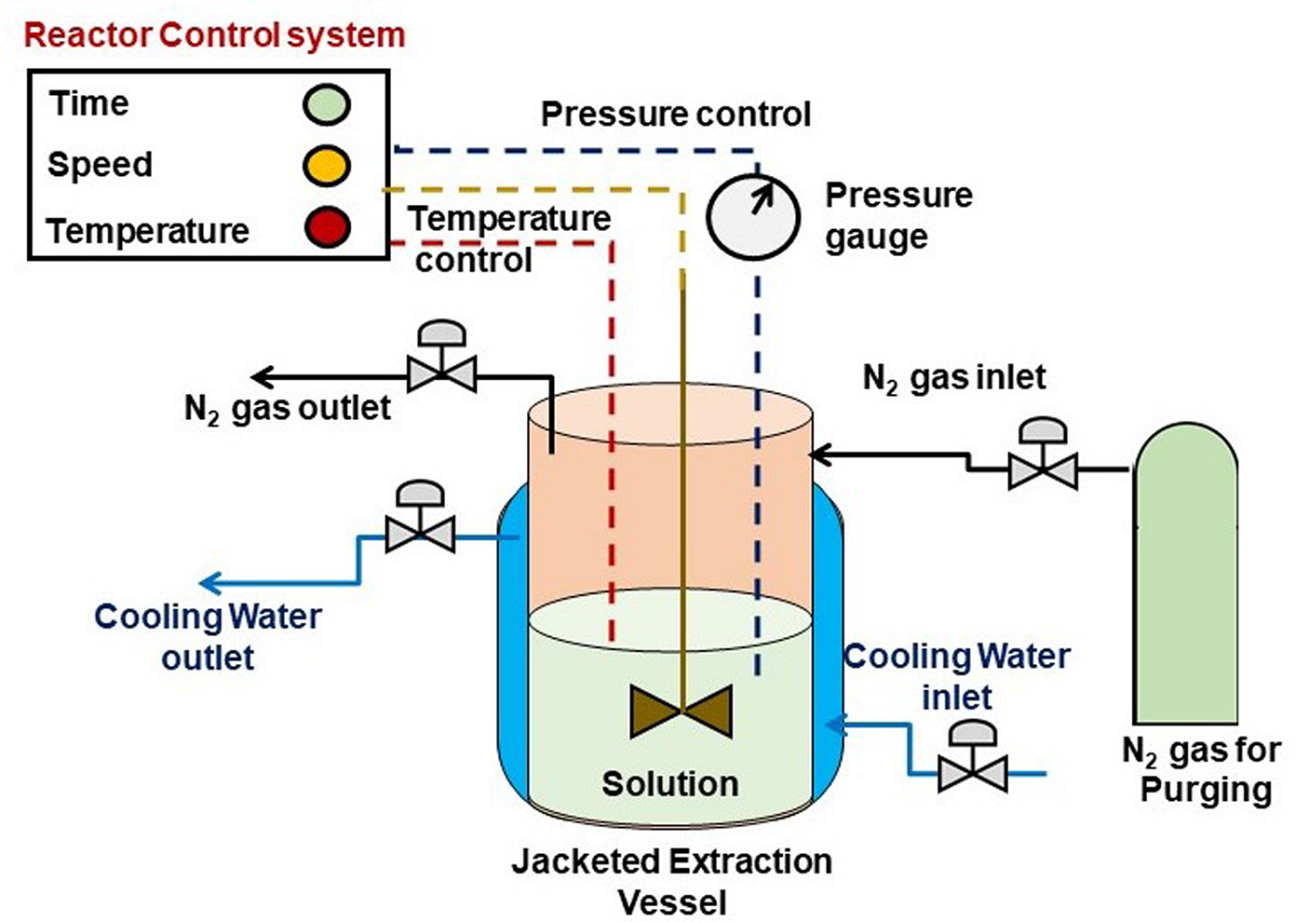
Diagram of subcritical solvent extraction.

Subcritical solvent extraction (SSE), also known as hot solvent extraction or pressurized hot solvent extraction, is an innovative technique that utilizes a solvent (generally water) at temperatures below its critical point and pressures below its critical pressure. This method takes advantage of solvent’s unique properties under subcritical conditions, enhancing its ability to solubilize various bioactive compounds from natural sources. A schematic diagram of SSE is shown in Figure 6.

The experimental procedure involves several crucial steps. First, the natural sources are loaded into an extraction vessel. The solvent is added, and the system is pressurized to subcritical conditions, maintaining the solvent in a liquid state. This unique state of solvent enhances its solvent properties, allowing it to penetrate the solid matrix effectively. During the extraction process, the subcritical liquid acts as a solvent, solubilizing bioactive compounds from natural sources. The raised temperature and pressure contribute to increased mass transfer, accelerating the extraction kinetics. One significant advantage is the selectivity of SSE, targeting specific compounds without degrading thermally sensitive bioactives. The extraction duration is relatively short compared to traditional methods. During subcritical solvent extraction, separation of the product from the solvent typically occurs through a process called phase separation or solvent recovery. This method takes advantage of the differences in densities between the product and the solvent. After the extraction process, the mixture of product and solvent is cooled, allowing the product to precipitate or separate out from the water solvent. The product can then be collected through filtration, centrifugation, or other separation techniques, while the water solvent can be recovered and recycled for further use in the extraction process. This separation ensures that the extracted product is isolated from the solvent, allowing for further purification or analysis as needed. The resulting extract is enriched with bioactive components and is typically free from organic solvents. SSE is considered an eco-friendly, efficient, and selective method for obtaining bioactive compounds from natural sources, making it a valuable approach in the field of green extraction technologies.

#### 2.4.1 Key Parameters for Efficiency and Yield Enhancement in SSE

##### 2.4.1.1 Temperature

Temperature significantly stimuluses SSE by altering solvent’s properties. Raised temperatures enhances solvent’s solubility and diffusivity, promoting efficient compound extraction. However, excessive temperatures may lead to degradation of heat-sensitive components. In the study on subcritical butane extraction of acer truncatum seed oil, the effect of temperature on extraction yield was investigated by Wang et al.^42^ The extraction yield was found tobe increases with an increase in temperature from 20 to 60 °C. The maximum yield of 47.22 wt % was obtained at an optimum temperature of 50 °C, beyond which it remained constant. The enhanced extraction yield with temperature rise was attributed to intensified thermal movement of molecules, reduced viscosity of acer truncatum seed oil, and improved convective diffusion. However, higher temperatures led to increased solvent evaporation, higher saturated vapor pressure, and potential damage to heat-sensitive nutrients in raw oil. The optimal temperature was determined to be 50 °C, where the extraction efficiency reached 94.50% after two extractions. The positive enthalpy change (20.06 kJ/mol) indicated an endothermic process, while the negative Gibbs’ free energy change confirmed the spontaneity of the extraction, supporting the preference for higher temperatures.^42^ In the study by Okoro et al.^43^ on insect farming waste valorization, the effect of temperature on subcritical water extraction for lipid recovery was investigated. An increase in temperature from 150 to 200 °C resulted in increase in lipid yield from 6.13 to 8.68 wt %, attributed to changes in water polarity, diffusion rate, and viscosity. However, a subsequent decrease to 7.72 wt % at 250 °C was observed, likely due to thermally induced lipid splitting. The study emphasized the importance of temperature control in optimizing lipid extraction efficiency. The optimized subcritical water extraction conditions, determined as 236.8 °C, yielded an enhanced lipid content of 13.31 wt %, contributing to the development of a sustainable biodiesel production process from insect farming waste. However, in the subcritical butane extraction of hermetia illucens as studied by Chen et al.,^44^ the temperature significantly influenced the oil yield. The yield was found to be increased from 27.5% to 29.5% upon increasing the temperature from 25 to 55 °C but thereafter it slightly reduced at 65 °C. The excessive temperature may lead to solvent volatilization and retrograde solubility, reducing oil solubility. The optimized temperature range, balancing production cost and oil yield, was determined to be 35 to 55 °C. Finding the optimal temperature is crucial for maximizing extraction efficiency while minimizing energy consumption and preserving the integrity of the extracted compounds. Careful control of temperature in subcritical solvent extraction ensures the selective and efficient recovery of desired components from various substrates.

##### 2.4.1.2 Extraction Time

Extraction time is a critical parameter in SSE, determining the duration of contact between solvent and target compounds. Adequate time allows for the dissolution of compounds into the solvent, ensuring optimal extraction efficiency. In the subcritical butane extraction of black hermetia illucens, as studied by Chen et al.,^45^ the extraction time played a crucial role in oil yield ranging from 15 to 80 min. Single-factor experiments were conducted at a constant temperature of 45 °C, and solid-to-solvent ratio of 1:2 g/mL. The results demonstrated a general increasing trend in oil yield with longer extraction times, ranging from 28.96% to 29.64% on increasing time from 20 to 60 min. The diminishing returns suggest that after a certain point, the diffusive movement of butane reaches a dynamic equilibrium, and further extraction time does not significantly enhance oil yield. The optimal extraction time, as determined by response surface methodology, ranged from 30 to 50 min. This finding provides valuable insights into the temporal dynamics of extraction for maximizing black soldier fly larvae oil yield.^44^ Whereas in the study on insect farming waste valorization, the effect of time during subcritical water extraction for lipid recovery was investigated.^43^ The optimized SSE conditions for enhanced lipid yield were determined as 236.8 °C temperature, 10 min of extraction time, water as solvent, and 1 g/100 mL solid loading. The study observed a negative correlation between increasing extraction times and lipid yield, emphasizing the impact of sustained heating on the thermal stability of lipids. Prolonged extraction times, beyond 15 min, were found to decrease lipid yield due to the potential degradation of lipid molecules. The optimized conditions resulted in a lipid yield of 13.31 wt %.^43^ The smaller time of extraction may result in incomplete extraction, while excessively long times could lead to unnecessary energy consumption and degradation of thermally sensitive compounds. Balancing extraction time is essential for achieving maximum yields and maintaining process efficiency.

##### 2.4.1.3 Solid to Solvent Ratio

An appropriate ratio ensures sufficient contact between the solid matrix and water, allowing for optimal dissolution of target compounds. In the subcritical butane extraction of acer truncatum seed oil, the effect of the solvent-to-solid ratio on extraction yield was investigated by Wang et al.^42^ The study explored ratios ranging from 2:1 to 6:1 (mL/g). The initial ratio was set to 2:l for complete seed powder immersion, and the extraction yield increased with the ratio, peaking at a slightly higher yield when the ratio exceeded 3:1. As the solvent amount increased, the contact area between seed powder and solvent expanded, leading to a higher mass transfer driving force, favoring extraction. However, beyond a certain point, additional solvent yielded diminishing returns. The solvent to solid ratio, balancing efficiency and cost considerations, was determined to be 3.4:1. This ratio, along with optimized time and temperature conditions, resulted in a high extraction efficiency of 94.50% after two extraction cycles under subcritical butane conditions. A higher ratio may increase the contact area but could lead to increased costs and energy consumption. Conversely, a lower ratio might limit extraction efficiency. Balancing the solid-to-solvent ratio is crucial for achieving maximum yields and cost-effective SSE processes. Summary of some of the SSE extraction investigations documented in the literature are given in Table 1.

#### 2.4.2 Merits of SSE

SSE typically uses water as the solvent, eliminating the need for organic solvents that may be harmful to the environment. This makes the process more sustainable and reduces the generation of hazardous waste. Solvents (water, ethanol, butane, acetone, etc.) used for SSE are relatively low-cost solvents and are readily available, making SSE more energy-efficient compared to some traditional extraction methods that require the use of hazardous, toxic, and expensive organic solvents. SSE has enhanced solvent properties due to its higher temperature and pressure, leading to faster extraction times compared to conventional methods. This can result in higher efficiency and productivity. The selectivity of SSE can be altered by adjusting temperature and pressure conditions. This allows for the targeted extraction of specific compounds while leaving others behind, providing a degree of control over the process: Since water is used mainly as the extraction solvent, there is generally a lower risk of residual solvents in the extracted material, making the final product safer and more suitable for applications in food and pharmaceutical industries. SSE is versatile and can be applied to a wide range of compounds, including polar and nonpolar substances. This makes it suitable for extracting various types of analytes.

#### 2.4.3 Limitations of SSE

While subcritical solvents have good solvating properties, they may not be effective for extracting certain types of compounds with limited solubility. This limitation can affect the efficiency of the extraction process. SSE typically requires higher temperatures and pressures, which may impose constraints on the materials of construction for the extraction equipment. This can lead to higher costs and potential safety concerns. Some compounds may be thermally sensitive and prone to decomposition at the higher temperatures used in SSE. This can result in the loss of target compounds or the formation of undesirable byproducts. The design and construction of equipment capable of handling high temperatures and pressures can be complex and expensive. This may pose challenges, particularly for small-scale applications or laboratories with limited resources.

#### 2.4.4 Recent Advances in SSE

Hybrid extraction, SSE with other methods like SFE, presents a breakthrough in bioactive compound extraction. This approach enhances extraction efficiency, reduces environmental impact, and maintains the biological activity of extracted compounds. SSE’s ability to alter solvent properties under high temperature and pressure, combined with techniques like SFE, offers a versatile and sustainable extraction process. The hybrid extraction process, combining SSE and SFE, demonstrates significant advancements in the production of 5-hydroxymethyl furfural from fructose by Oriez et al.^45^ Subcritical water extraction plays a pivotal role in the discussed study by compensating for water coextraction during supercritical carbon dioxide (scCO_2_) extraction, thereby maintaining the volume of the reaction mixture. Despite potentially diluting the reaction medium, continuous injection of water ensures the integrity of the aqueous phase, crucial for preventing degradation of the target compound, 5-hydroxymethyl furfural (HMF). This method allows for the achievement of a remarkable HMF maximum yield of 62.4%, alongside exceptional separation efficiency (97.3%) and high relative purity (95.8 wt %) in the extract. Furthermore, subcritical water extraction enables the avoidance of postreactional purification steps, reducing the overall production cost of HMF. By providing a stable reaction environment and facilitating efficient extraction, subcritical water contributes significantly to the success of the extractive reaction process, offering a sustainable and economically viable approach for HMF production. Notably, 5-hydroxymethyl furfural is achieved with a remarkable 62.4% yield at 160 °C and 25 MPa, for 420 min. The hybrid extraction process holds promise in enhancing efficiency and purity while minimizing the environmental impact of bioactive compound extraction.^45^ The synergy of these methods addresses challenges in yield, biological activity, and environmental sustainability, making hybrid extraction a valuable advancement for industries seeking efficient and eco-friendly extraction of bioactive compounds from natural sources.

The semicontinuous approach in SSE holds paramount importance due to its efficiency in processing large volumes of biomass continuously. In this method, the sample is introduced continuously, enabling a more efficient and continuous extraction process. The semicontinuous subcritical water extraction method refers to a process where water flows continuously through the extraction system, allowing for the extraction of valuable compounds from various biomasses in a scalable and economically viable manner. Solvent at subcritical conditions promoting the extraction of target compounds from the sample. This approach allows for a steady extraction process with reduced batch-to-batch variability. It is particularly beneficial for large-scale operations, providing a balance between the benefits of continuous processing and the advantages of SSE in maintaining the integrity of heat-sensitive compounds. The study on semicontinuous flow through subcritical water hydrolysis of grape pomace (*Vitis vinifera* L.) by Castro et al.,^46^ represents a significant advancement in subcritical water extraction. The exploration of pH (6–10) and temperature (150–210 °C) revealed optimal conditions for recovering sugars and organic acids. The highest sugar concentration of 34.64 g/100 g was achieved at pH 6 and 150 °C, emphasizing the importance of pH control in the process. The temperature (210 °C) at pH 10 yielded low inhibitory compounds (1.59 g/100 g) and high organic acid content (290.76 g/100 g). The study demonstrated a substantial hemicellulose reduction of approximately 75% through thermogravimetric analysis, highlighting the structural modification of biomass during subcritical hydrolysis. Importantly, the study provides a comprehensive understanding of operational conditions for optimizing sugar (150 °C and pH 6) and organic acid (210 and pH 10) recovery, with key numerical values defining the process efficiency. This research underscores subcritical water’s potential as a sustainable method for valorizing grape pomace by extracting valuable compounds.^46^ This method ensures consistent product quality while optimizing resource utilization. The semicontinuous flow design enhances productivity, reduces processing time, and facilitates better control over reaction conditions. This approach is particularly significant in the extraction of valuable compounds from various biomasses, offering scalability and economic viability for industries using a semicontinuous approach.

### 2.5 Solid-Phase Microextraction (SPME)

**Figure 7:**
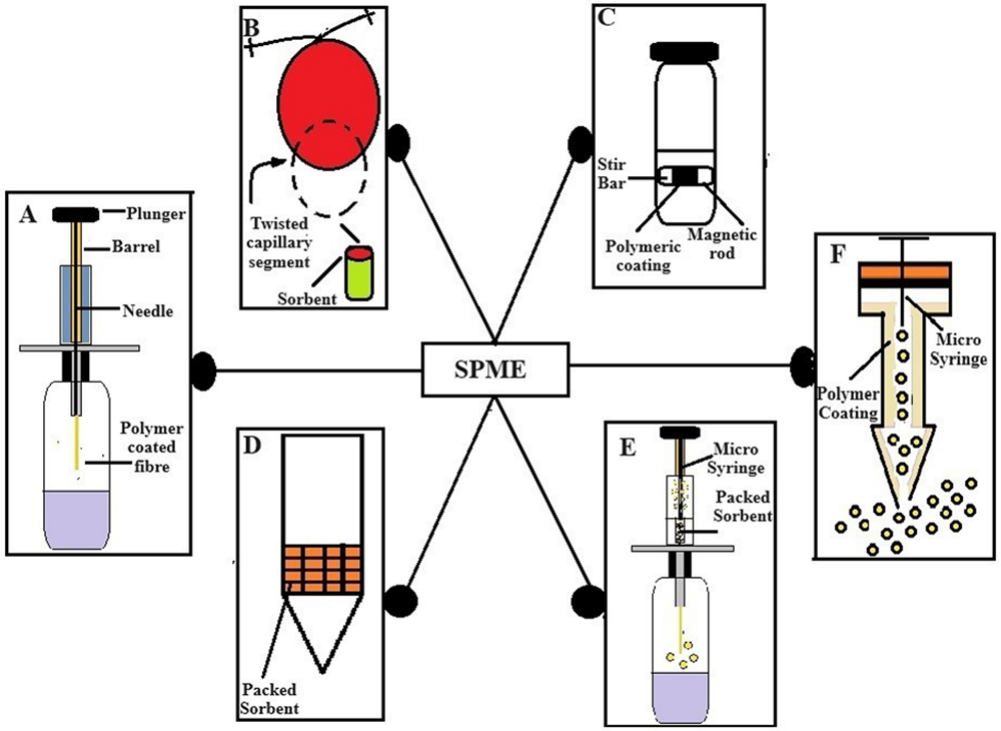
Schematic diagram of solid-phase microextraction (A) Direct SPME (dSPME) (B) Twist SPME (Tw-SPME) (C) Stir bar sorptive extraction (SBSE) (D) BioSPME (E) (F) In-Tube SPME (IT-SPME).

Solid-phase microextraction (SPME) is a technique used for the extraction and concentration of target analytes from various matrices. It is a solvent-free method that employs a solid-phase fiber coated with a thin layer of appropriate sorbent material. Solid-phase microextraction (SPME) is advantageous for extracting a diverse array of bioactive compounds from micro to nano concentrations. These include volatile organic compounds (VOCs), polyaromatic hydrocarbons (PAHs), pesticides, pharmaceuticals, endocrine disruptors, flavonoids, phenolic compounds, amino acids, and peptides.^53−55^ Examples of costly bioactive components in the pharmaceutical industry include certain peptides like insulin, which is essential for managing diabetes, and monoclonal antibodies such as trastuzumab used in cancer treatment.^61−63^ These compounds often require intricate extraction and purification processes, and their high demand and specialized production contribute to their elevated costs in pharmaceutical products. A schematic diagram of SPME is shown in Figure 7. In SPME, the fiber is exposed to the sample, allowing the analytes to partition between the sample matrix and the sorbent coating on the fiber.^52^ The analytes are adsorbed onto the sorbent, concentrating them for subsequent analysis. The advantages of SPME include its simplicity, rapidity, and versatility. It eliminates the need for extensive sample preparation steps and large volumes of organic solvents. SPME can be applied to various sample types, including liquids, gases, and solid surfaces.^53^ The SPME fiber is conditioned before use to remove any contaminants and ensure reproducible extraction performance. The fiber is exposed to the sample matrix, allowing the analytes to partition between the sample and the sorbent coating on the fiber. This can be done by immersing the fiber directly into the sample or by exposing it to the headspace above the sample. The fiber remains in contact with the sample for a specific equilibration time, allowing the analytes to reach equilibrium between the sample matrix and the fiber coating. After equilibration, the fiber is removed from the sample and transferred to the analytical instrument for desorption. This is typically done by placing the fiber into the injection port of a gas chromatograph (GC) or directly immersing it into a liquid chromatograph (LC) for desorption and subsequent analysis. The desorption process involves releasing the absorbed analytes from the sorbent coating so that they can be analyzed.

There are several variations of SPME methods as shown in Figure 7, each tailored to specific applications and analytical requirements. Some common types of SPME methods include: **A. Direct SPME (dSPME):** This is the standard SPME method where the coated fiber is directly exposed to the sample for extraction. After extraction, the fiber is transferred to the analytical instrument for desorption and analysis. **B. Twist SPME (Tw-SPME):** In this method, the SPME fiber is twisted to increase the exposed surface area, enhancing the extraction efficiency. **C. Stir bar sorptive extraction (SBSE):** While not technically SPME, SBSE is a related technique where a magnetic stir bar coated with a sorbent material is used for sample extraction. The stir bar is then placed in the sample for extraction, and the desorption is typically done using thermal or solvent desorption. **D. BioSPME:** In BioSPME, the SPME fiber is coated with a biologically active packed material, such as antibodies or molecularly imprinted polymers, for selective extraction of target analytes. **E. Headspace SPME (HS-SPME):** In this method, the SPME fiber is exposed to the headspace above a sample, allowing volatile compounds to partition into the fiber coating. It is commonly used for the analysis of volatile organic compounds in gas or liquid samples. **F. In-Tube SPME (IT-SPME):** This variation involves placing the SPME fiber inside a capillary column. Sample extraction occurs as the sample passes through the column, and the analytes are then desorbed for analysis. The choice of the SPME method depends on the nature of the analytes, the complexity of the sample matrix, and the analytical instrumentation used for subsequent analysis. Researchers often select the most suitable SPME method based on the specific requirements of their analytical application. SPME is widely used in various fields, including environmental analysis, food and beverage analysis, pharmaceutical analysis, and forensic applications. It has proven to be a valuable tool for the extraction and concentration of volatile and semivolatile organic compounds.

#### 2.5.1 Key parameters for efficiency and yield enhancement in SPME

##### 2.5.1.1 Extraction time

Time plays a crucial role in SPME as it directly influences the efficiency and kinetics of analyte extraction. The duration of extraction determines the amount of analyte absorbed by the sorbent coating, impacting the method’s sensitivity and detection limits. Zhang et al.,^52^ conducted a comprehensive study to develop a direct immersion solid phase microextraction (DI-SPME) for the determination of polycyclic aromatic hydrocarbons, polychlorinated biphenyls, and pesticide residues in edible seaweeds. The conditions for simultaneous determination included a buffer at pH 7.0, 20% acetone, 10% NaCl, 0.02% NaN_3_, 60 min extraction at 55 °C, and 20 min desorption at 270 °C. Notably, the study highlighted the significance of binding time, emphasizing variations in extraction results at different time intervals (10–80 min) postsample preparation. Similarly, an investigation by Letseka et al.,^53^ explored the impact of extraction time on a combined liquid phase microextraction and dispersive liquid–liquid microextraction method for hexestrol and atrazine in aqueous systems. The optimized conditions featured toluene in the acceptor phase, a 1:1 chloroform: toluene (v/v) mixture as the dispersed solvent, 15% NaCl, and a 15 min extraction time. This short extraction time represented a significant advancement in extraction kinetics compared to traditional systems. The method achieved notable enrichment factors (concentration of the analyte in the extracted/enriched sample to its concentration in the original sample) of 87 and 62 fold for atrazine and hexestrol, respectively, low detection limits (lowest concentration or amount of an analyte in a sample that can be reliably detected but not necessarily quantified) of 0.072 ng/mL and 0.063 ng/mL for atrazine and hexestrol, respectively, excellent linearity (R^2^ > 0.9959), and acceptable repeatability (%RSD (relative standard deviation) < 11%). Optimal extraction time is essential for achieving maximum analyte recovery while minimizing the risk of saturation or desorption. Understanding the temporal aspects of SPME ensures the development of efficient, reproducible methods for various analytes and sample matrices.^53^ Optimizing extraction time contributes to robust SPME protocols, enhancing the technique’s versatility and reliability in applications ranging from environmental monitoring to food safety analysis.

##### 2.5.1.2 Sorbent

The sorbent in SPME plays an essential role as it precisely determines the extraction efficiency, sensitivity, and selectivity of the technique. It serves as the medium for capturing and concentrating target analytes from the mixture. In the study by Maria et al.,^54^ a novel generation of silver-based polymeric ionic liquid sorbent coatings for SPME was introduced. These coatings, derived from ionic liquid monomers with silver ions, exhibited enhanced thermal stability, allowing effective thermal desorption of analytes at 175 °C. The silver-based polymeric ionic liquid sorbent coating demonstrated effective detection, with limits ranging from 2.6 to 8.2 mg/L in ultrapure water. The best-performing polymeric ionic liquid showed consistent results with relative standard deviations below 13% at a spiked level of 160 mg/L. The method successfully analyzed rinsewater from a dairy farm, detecting analytes at concentrations between 52 and 179 mg/L, thus validating their practical utility. Similarly, In the study by Zheng et al.,^55^ they synthesized magnetic sorbent using magnetic nanoparticles (Fe3O4@SiO_2_–C_18_ NPs), significantly impacted the extraction efficiency of endogenous volatile organic metabolites by SPME. Optimization experiments revealed that 40 mg of sorbent was optimal for extraction, and adsorption was time-dependent, reaching an optimum at 60 min. Elution time, however, showed no time dependency, with 30 min being the optimal elution time. The method exhibited a limit of detection ranging from 9.7 to 57.3 ng/mL, a limit of quantification from 32.4 to 190.9 ng/mL, recoveries from 42.6 vol % to 99.1 vol %, and good precision with intra- and interday standard deviation values below 3% and 11%, respectively.^55^ Overall, these studies showcase the development and application of advanced sorbent materials for efficient and sensitive analytical techniques. The sorbent plays a crucial role in the dispersive micro solid-phase extraction procedure for extracting organophosphorus pesticides in various samples.^55^ Similarly, the optimization of sorbent composition, including metal–organic framework, chitosan, magnetic Fe_3_O_4_ nanoparticles, and silica nanoparticles, was performed using a simplex lattice mixture design by Ghorbani et al.,^56^ for vegetable, fruit juice, and milk samples. The obtained quadratic equation indicated that the optimized sorbent composition percentages were 19.60% Fe_3_O_4_, 37% Chitosan, 10% SiO_2_, and 33.40% ZIF-67, resulting in a response of 83.01%. The sorbent optimization, guided by statistical analysis, led to a highly efficient extraction system. The choice of a suitable sorbent is critical for enhancing the method’s performance, allowing for efficient extraction and desorption of analytes. Tailoring sorbents to specific applications contributes to the success of SPME in various fields, including environmental monitoring, pharmaceutical analysis, and bioanalytical studies.^56^ Overall, the sorbent’s importance lies in its ability to improve the sensitivity and reliability of SPME for diverse analytical challenges.

##### 2.5.1.3 Solid liquid ratio

The solid–liquid ratio in SPME plays a crucial role in determining the concentration of analytes extracted from a sample. It represents the amount of solid-phase sorbent relative to the sample volume, impacting extraction efficiency. In the presented study by Tuzen et al.,^57^ the solid-to-solvent ratio, a critical parameter in the extraction process, has a direct bearing on the efficiency of the developed method for thiabendazole analysis by SPME. The extraction efficiency was assessed by varying the ratio of glycolic acid and betaine mixture (1:2 v/v) in the zwitterionic deep eutectic solvent. The method’s efficacy was investigated by altering the solvent volume up to 900 μL. The optimal recovery values were achieved using 600 μL of deep eutectic solvent, emphasizing the importance of a balanced solid-to-solvent ratio for quantitative thiabendazole recoveries. For optimal results, consistent recovery of 75.1%, 62.9%, and 84.4% (v/v) was achieved for different acid-betaine complexes 2-furoic acid, phenylacetic acid, and mandelic acid, respectively. The study by Letseka et al.,^53^ investigated the impact of the liquid ratio, specifically the ratio of chloroform to toluene (v/v), on the efficiency of a combined liquid phase microextraction and dispersive microextraction method for extracting hexestrol and atrazine from aqueous systems. The optimized conditions included toluene in the acceptor phase, a 1:1 chloroform: toluene (v/v) mixture as the dispersed solvent, 15% NaCl, and a 15 min extraction time. The examination of different ratios (0.2:1–2:1 v/v) of chloroform to toluene revealed that the extraction efficiency increased with an increasing ratio, peaking at 1:1. However, efficiency decreased beyond this ratio. Notably, a maximum volume of 25 mL for the 1:1 chloroform:toluene mixture was determined to prevent sedimentation. The method achieved enrichment factors of 87 and 62 fold for atrazine and hexestrol, respectively. The detection limits were 0.018 and 0.016 mg/mL using flame ionization detection, and 0.072 ng/mL and 0.063 ng/mL using single ion monitoring mass spectrometry for atrazine and hexestrol, respectively. The study highlights the importance of optimizing the liquid ratio for achieving optimal extraction efficiency.^53^ An optimal solid–liquid ratio ensures that the sorbent can efficiently interact with target analytes, enhancing sensitivity and reproducibility. Too high a ratio may lead to incomplete analyte extraction, while too low a ratio could result in insufficient sensitivity.^57^ Precise control of the solid–liquid ratio is essential for maximizing the extraction performance of SPME, making it a critical parameter in sample preparation for various analytical applications.

##### 2.5.1.4 Solvent

The selection of solvent in solid-phase microextraction (SPME) is crucial for optimizing the extraction process. It directly impacts extraction efficiency, selectivity, and desorption characteristics, influencing the accuracy and sensitivity of subsequent analyses. In the development of a vortex-assisted dispersive microextraction method for thiabendazole in fruit samples by Tuzen et al.,.^57^ The choice of solvent, particularly the zwitterionic deep eutectic solvent (Zw-DES), plays a crucial role in the efficacy of the extraction method. By mixing betaine with various acids, including 2-furoic acid, phenylacetic acid, mandelic acid, and glycolic acid, Zw-DESs with different properties were prepared. These solvents were then utilized for the extraction of thiabendazole (TBZ) from fruit samples. The efficiency of the extraction process was evident in the achieved analytical parameters as broad linear range (0.4–150 μg/L) high preconcentration factor (150), and low relative standard deviation (below 2.5%). Moreover, the Zw-DES-based method exhibited ecological safety, ease of extraction, and biodegradability, making it a green and effective approach for TBZ determination in fruit samples. the choice of extraction solvent significantly influenced efficiency. Four zwitterionic deep eutectic solvents were tested, revealing the superiority of a glycolic acid and betaine mixture (1:2 v/v) with recoveries of 75.1%, 62.9%, and 84.4% (vol) for 2-furoic acid, phenylacetic acid, and mandelic acid, respectively. The unique properties of Zw-DES, such as its ecological compatibility, low melting point, and thermal conductivity at room temperature, contribute to its superiority in microextraction. Additionally, Zw-DES, being a natural and renewable compound, adds to its appeal for analytical applications, ensuring the safety of food samples by accurately quantifying TBZ residues. In the presented study by Ghorbani et al.,^56^ the choice of desorption solvent significantly influenced the efficacy of the dispersive micro solid-phase extraction procedure for vegetable, fruit juice and milk samples. Methanol was identified as the optimal desorption solvent, displaying the highest peak area for analyte determination. The quantitation limits and detection limits were impressively low, below 0.38 ng/mL and 0.11 ng/mL, respectively. Moreover, the relative standard deviations were less than 4.59%. This optimized approach showcases the importance of solvent selection in enhancing extraction efficiency and sensitivity in complex matrices.^56^ However, the study by Yurt et al.,^58^ investigated the effect of hydrogen-enriched solvents on the extraction of phytochemicals in propolis. Hydrogen-rich solvent systems, including hydrogen-rich water, hydrogen-rich ethanol, and hydrogen-rich methanol, were compared with their regular counterparts. Results indicated that hydrogen-enriched solvents enhanced the extraction efficiency of phenolic content and antioxidant activity in propolis. Specifically, hydrogen-rich water extraction was efficient in improving total phenolics and antioxidant activity measured by the ABTS method. On the other hand, hydrogen-rich ethanol and hydrogen-rich methanol showed higher results for total flavonoids and antioxidant activity measured by the DPPH method. These findings suggest that the choice of hydrogen-enriched solvent could impact the extraction efficiency of specific phytochemicals in propolis. However, the study recommends further extensive research to fully understand the effect of hydrogen-enriched solvents on phytochemical extraction efficiency. Additionally, safety considerations need to be addressed before widening the application of hydrogen-enriched solvents, especially in the food and nutraceutical industries. This study provides valuable insights into optimizing solvent choice for phytochemical extraction, potentially enhancing the quality and efficacy of propolis-derived products. A well-chosen solvent enhances the solubility of target analytes, minimizes matrix effects, and ensures compatibility with analytical techniques. Additionally, considering environmental sustainability and the versatility of the solvent contributes to the overall significance of its selection, enabling efficient extraction across diverse sample matrices and analyte types in a reproducible manner.

##### 2.5.1.5 Temperature

Temperature is a critical parameter in SPME that exerts a direct effect on the rate of analyte sorption and desorption onto/from the sorbent coating. Controlling temperature allows optimization of extraction efficiency, affecting sensitivity and selectivity. Higher temperatures can enhance mass transfer, accelerating analyte absorption, while lower temperatures facilitate desorption during analysis. In the study by Zhang et al.,^52^ temperature played a critical role in the efficiency of the direct immersion-solid phase microextraction-gas chromatography-mass spectrometry (DI-SPME-GC-MS) method for the determination of contaminants in edible seaweeds. The optimized extraction temperature varied based on the hydrophobicity of the analytes, For lipophilic compounds (Log*P* > 5.6): The optimum extraction temperature was 80 °C, which resulted in enhanced response for highly lipophilic compounds. For medium lipophilicity compounds (3.46 < LogP < 5.6): The extraction temperature was set at 30 °C, favoring the extraction of compounds with moderate hydrophobicity. For hydrophilic compounds (Log*P* < 3.46): A lower extraction temperature of 30 °C was chosen to facilitate the extraction of hydrophilic compounds. Additionally, it was observed that some pesticides, like cypermethrin and cyfluthrin, showed decreased extraction efficiency at higher temperatures due to compound degradation. Thus, for hydrophobic analytes with Log*P* > 5.2, a lower extraction temperature of 55 °C was deemed more suitable to prevent degradation while still ensuring efficient extraction. These temperature variations were crucial in achieving optimal extraction efficiency and sensitivity for the different classes of analytes present in the seaweed samples, ensuring accurate determination of contaminants in the studied matrix. Understanding the thermal behavior of SPME enables the development of robust methods for diverse analytes and sample matrices, making temperature a key factor in maximizing the performance and applicability of SPME in fields such as environmental monitoring and chemical analysis. A summary of the recent SPME investigations documented in the literature are given in Table 1.

#### 2.5.2 Advantages of SPME

SPME is a relatively simple and user-friendly technique that requires minimal sample preparation. It eliminates the need for multiple steps such as liquid–liquid extraction or solid-phase extraction.^54^ SPME allows for rapid extraction and concentration of analytes from the sample matrix. The process can often be completed in minutes, providing quick results compared to traditional extraction methods.^55^ SPME is versatile and can be used for a wide range of sample types, including air, water, and solid samples. It is applicable to various analytes, such as volatile and semivolatile organic compounds.^55^ SPME can achieve high sensitivity because it concentrates analytes onto a small volume of the stationary phase, leading to improved detection limits in analytical methods.^56^ SPME allows for in situ sampling, enabling the extraction of analytes directly from the sampling environment. This is particularly useful for on-site analysis or monitoring applications.^56^ Extracted analytes from SPME can be directly introduced into gas chromatography (GC) or liquid chromatography (LC) systems, simplifying the analytical process and reducing the need for additional sample handling steps.^57^

#### 2.5.3 Limitations of SPME

SPME has limitations in terms of sample volume that can be processed. For larger sample volumes, other extraction techniques may be more suitable.^52^ SPME is more effective for low to moderate molecular weight compounds. High molecular weight analytes may have limited affinity for the SPME fiber, affecting the extraction efficiency.^59^ The presence of complex sample matrices can lead to matrix interferences, affecting the extraction efficiency and selectivity of SPME. Sample cleanup steps may be necessary for certain applications.^59^ SPME fibers can degrade over time due to repeated use or exposure to certain sample matrices. Contamination from previous analyses may also occur, affecting the reliability of results.^59^ The dynamic range of SPME may be limited for certain analytes, especially when dealing with samples containing a wide range of concentrations.^60^ SPME relies on reaching equilibrium between the analyte in the sample and the stationary phase on the fiber.^60^ This equilibrium time can vary, and longer equilibration times may be needed for some applications. Despite these limitations, SPME remains a valuable and widely used technique in analytical chemistry, particularly for its simplicity, speed, and efficiency in a variety of applications. Careful consideration of its strengths and weaknesses is essential for optimizing its use in specific analytical scenarios.

#### 2.5.4 Recent Advancements in SPME

High-throughput applications in SPME involve the simultaneous processing of numerous samples, enhancing efficiency and speed. In SPME, this approach finds application in parallel extractions, enabling swift analysis of large sample sets. High throughput applications in solid-SPME have revolutionized analytical chemistry by enhancing efficiency and sensitivity in sample analysis. The study by Carasek et al.^59^ highlights recent developments in microextraction, emphasizing high-throughput applications. Innovations include a nanocomposite of titania hydroxyapatite in a 96 fiber array for parallel extraction of doxorubicin, exhibiting 86.1% to 112.3% accuracy. A multifiber device, featuring distinct molecularly imprinted polymers, achieves efficient extraction of organophosphorus with 75.1% to 123.2% accuracy. The SPME brush with 96 stainless-steel pins, coated with hydrophilic–lipophilic balance particles and polyacrylonitrile, quantifies over 100 veterinary drugs in chicken and beef tissue, demonstrating excellent accuracy and precision. Ambient ionization techniques like DART-MS/MS (direct analysis in real time tandem mass spectrometry, a technique used in mass spectrometry for the rapid analysis of samples with minimal or no sample preparation) and paper spray coupled with SPME enable rapid screening of 98 analytes in bovine tissue.^59^ Microfluidic open interface coupled with SPME achieves simultaneous extraction of 96 plasma samples for therapeutic drug monitoring. These advancements showcase the potential of high-throughput microextraction techniques in achieving efficient, automated, and environmental friendly sample preparation for diverse analytical applications.^59^ High-throughput SPME methods, coupled with automation and advanced technologies, cater to the demand for rapid results and contribute to diverse fields, such as environmental monitoring, pharmaceuticals, and food analysis. These applications underscore the role of high-throughput SPME in streamlining sample preparation for various analytical purposes. High throughput SPME ensures swift identification of target compounds, enabling timely decision-making in research and industry. The technique’s importance extends to improving overall analytical workflows, increasing sample throughput, and advancing our understanding of complex sample matrices.^59^

Nanotechnology revolutionizes SPME, enhancing its analytical prowess. Nanostructured materials in SPME exhibit increased surface area and tailored properties, amplifying adsorption capacities for precise molecule detection. Functionalized nanomaterials heighten selectivity, targeting specific analytes, while miniaturization enables portable, on-site analysis. This amalgamation propels SPME to new heights, offering unparalleled sensitivity, efficiency, and versatility in monitoring environmental polycyclic aromatic hydrocarbons, thereby advancing analytical capabilities and fostering practical applications in diverse fields. In the study by Piryael et al.,^60^ the synthesis of MnMoO4/NiCO_2_O_4_ on a graphitized pencil lead for extraction of polycyclic aromatic hydrocarbons in surface water samples showcases the potential of nanomaterials in creating high-performance SPME fibers. The significance of nanotechnology in SPME, as demonstrated in the presented study, lies in its ability to enhance the efficiency and sensitivity of polycyclic aromatic hydrocarbons measurements in surface water samples. The resulting fiber exhibits exceptional adsorption power, with a substantial increase in surface area and unique morphology, contributing to improved adsorption capabilities for polycyclic aromatic hydrocarbons. The optimized conditions yield a linear response in the concentration range of 0.001 to 10 mg/L, with correlation coefficients (*R*^2^) ranging from 0.998 to 0.983 and limits of detection (LOD) between 0.2 and 3.8 ng/L.^59^ This signifies the precise and sensitive nature of the nanotechnology-enhanced SPME method, offering a solvent-free, highly selective, and low-detection-limit approach for environmental monitoring of polycyclic aromatic hydrocarbons in surface water samples. The study’s findings underscore the pivotal role of nanotechnology in advancing analytical techniques for accurate and efficient pollutant detection in the environment.^60^ The integration of nanotechnology into SPME also facilitates miniaturization, enabling the development of portable and efficient devices for on-site analysis. Overall, the synergy between nanotechnology and SPME enhances the precision, sensitivity, and versatility of analytical methods, making significant strides in the monitoring and measurement of pollutants in various complex sample matrices.

### 2.6 Future Scope for Advanced Extraction Methods

The future scopes for advanced extraction methods are diverse and promising, with ongoing research and technological advancements shaping the landscape of extraction science. Some key areas of future development include **Green and sustainable extraction:** Increasing focus on environmentally friendly extraction methods, utilizing eco-friendly solvents, and reducing energy consumption and integration of renewable energy sources and green technologies to make extraction processes more sustainable. **Process optimization and automation:** Continued optimization of extraction parameters using artificial intelligence, machine learning, and optimization algorithms and implementation of automated systems for enhanced reproducibility and high-throughput extraction. **Hybrid extraction technologies:** Exploration of hybrid extraction methods combining multiple techniques for synergistic effects and integration of complementary methods, such as coupling traditional solvent-based extraction with innovative technologies like ultrasound, microwaves, or supercritical fluids. **Customization for specific industries:** Tailoring extraction methods to meet the specific needs of industries such as pharmaceuticals, nutraceuticals, food and beverage, and cosmetics. Customized extraction protocols for specific bioactive compounds relevant to each industry. **Advancements in equipment design:** Development of advanced extraction equipment with improved scalability, modularity, and adaptability to various sample matrices. Integration of in-line monitoring and control systems for real-time process adjustments. **Biorefinery concepts:** Application of extraction methods in biorefinery processes for the efficient utilization of raw materials and byproducts. Integration of extraction with downstream processes to maximize the value extracted from natural sources. **Nanotechnology in extraction:** Exploration of nanomaterials for improved adsorption, separation, and enhanced extraction efficiency. Development of nanocarriers for targeted delivery of bioactive compounds extracted from natural sources. **Cross-disciplinary collaboration:** Collaborations between experts in chemistry, biology, engineering, and materials science to address complex challenges and unlock new possibilities. Integration of knowledge from diverse fields to create innovative and holistic approaches to extraction. **Regulatory compliance and standardization:** Establishment of standardized protocols for advanced extraction methods to ensure reproducibility and regulatory compliance. Development of guidelines and quality control measures for the industry.

The future of advanced extraction methods is dynamic, with ongoing efforts to make extraction processes more sustainable, efficient, and tailored to the specific requirements of various industries. The convergence of interdisciplinary research and technological innovations will continue to drive the evolution of extraction science in the coming years.

## 3 Summary and Conclusions

The review highlights the transformative landscape of advanced extraction methods for harnessing bioactive components from natural sources. The detailed exploration of diverse techniques, including supercritical solvent extraction, microwave-assisted extraction, ultrasound-assisted extraction, solid-phase microextraction, and subcritical solvent extraction, reveals a nuanced understanding of their applications and advantages. Supercritical solvent extraction emerges as a powerful and environmentally friendly method, utilizing supercritical fluids to achieve efficient extraction with minimal environmental impact. Microwave-assisted extraction offers rapid and selective extraction, optimizing both time and resource utilization. Ultrasound-assisted extraction proves effective through mechanical and thermal effects, enhancing mass transfer and extraction yields. Solid-phase microextraction showcases versatility, particularly in volatile compound extraction, providing a sensitive and rapid solution. Subcritical solvent extraction, operating under mild conditions, demonstrates promise for preserving thermolabile compounds. These advanced techniques collectively contribute to the evolving landscape of extraction methodologies, offering tailored solutions for diverse bioactive compounds and natural matrices. The integration of these methods into research and industrial practices promises enhanced efficiency, reduced environmental footprint, and increased selectivity. As the field advances, a nuanced understanding of the interplay between extraction parameters and compound characteristics will undoubtedly guide future innovations, fostering sustainable and optimized strategies for bioactive compound extraction from natural sources.

In conclusion, this review paper strongly advocates adopting advanced extraction techniques in research and industrial applications. The evidence presented unequivocally demonstrates their superiority in terms of efficiency and sustainability, unlocking new avenues for extracting valuable natural products while promoting responsible environmental practices. The transition to advanced methods is a scientific imperative and a practical choice for industries seeking to optimize their extraction processes, contributing to advancements in diverse fields and fostering eco-conscious practices. Researchers and practitioners are encouraged to embrace these advanced techniques as the future of natural product extraction.
